# Two-component spike nanoparticle vaccine protects macaques from SARS-CoV-2 infection

**DOI:** 10.1016/j.cell.2021.01.035

**Published:** 2021-03-04

**Authors:** Philip J.M. Brouwer, Mitch Brinkkemper, Pauline Maisonnasse, Nathalie Dereuddre-Bosquet, Marloes Grobben, Mathieu Claireaux, Marlon de Gast, Romain Marlin, Virginie Chesnais, Ségolène Diry, Joel D. Allen, Yasunori Watanabe, Julia M. Giezen, Gius Kerster, Hannah L. Turner, Karlijn van der Straten, Cynthia A. van der Linden, Yoann Aldon, Thibaut Naninck, Ilja Bontjer, Judith A. Burger, Meliawati Poniman, Anna Z. Mykytyn, Nisreen M.A. Okba, Edith E. Schermer, Marielle J. van Breemen, Rashmi Ravichandran, Tom G. Caniels, Jelle van Schooten, Nidhal Kahlaoui, Vanessa Contreras, Julien Lemaître, Catherine Chapon, Raphaël Ho Tsong Fang, Julien Villaudy, Kwinten Sliepen, Yme U. van der Velden, Bart L. Haagmans, Godelieve J. de Bree, Eric Ginoux, Andrew B. Ward, Max Crispin, Neil P. King, Sylvie van der Werf, Marit J. van Gils, Roger Le Grand, Rogier W. Sanders

**Affiliations:** 1 Department of Medical Microbiology, Amsterdam UMC, University of Amsterdam, Amsterdam Infection & Immunity Institute, 1105 AZ Amsterdam, the Netherlands; 2 Center for Immunology of Viral, Auto-immune, Hematological and Bacterial Diseases (IMVA-HB/IDMIT), Université Paris-Saclay, INSERM, CEA, Fontenay-aux-Roses, France; 3 Life and Soft, 92350 Le Plessis-Robinson, France; 4 School of Biological Sciences, University of Southampton, Southampton SO17 1BJ, UK; 5 Oxford Glycobiology Institute, Department of Biochemistry, University of Oxford, Oxford OX1 3QU, UK; 6 Department of Integrative Structural and Computational Biology, The Scripps Research Institute, La Jolla, CA 92037, USA; 7 Department of Viroscience, Erasmus Medical Center, 3015 GD Rotterdam, the Netherlands; 8 Department of Biochemistry, University of Washington, Seattle, WA 98195, USA; 9 Institute for Protein Design, University of Washington, Seattle, WA 98195, USA; 10 AIMM Therapeutics BV, 1105 BA Amsterdam, the Netherlands; 11 Molecular Genetics of RNA Viruses, Department of Virology, Institut Pasteur, CNRS UMR 3569, Université de Paris, Paris, France; 12 National Reference Center for Respiratory Viruses, Institut Pasteur, Paris, France; 13 Department of Internal Medicine, Amsterdam UMC, University of Amsterdam, Amsterdam Institute for Infection and Immunity, 1105 AZ Amsterdam, the Netherlands

**Keywords:** SARS-CoV-2, COVID-19, protection, antibodies, nanoparticles, vaccine, macaques, B cells, immunity

## Abstract

The severe acute respiratory syndrome coronavirus 2 (SARS-CoV-2) pandemic is continuing to disrupt personal lives, global healthcare systems, and economies. Hence, there is an urgent need for a vaccine that prevents viral infection, transmission, and disease. Here, we present a two-component protein-based nanoparticle vaccine that displays multiple copies of the SARS-CoV-2 spike protein. Immunization studies show that this vaccine induces potent neutralizing antibody responses in mice, rabbits, and cynomolgus macaques. The vaccine-induced immunity protects macaques against a high-dose challenge, resulting in strongly reduced viral infection and replication in the upper and lower airways. These nanoparticles are a promising vaccine candidate to curtail the SARS-CoV-2 pandemic.

## Highlights

• Two-component protein nanoparticles display multiple copies of the SARS-CoV-2 spike
• Spike protein nanoparticles enhance cognate B cell activation *in vitro*
• Vaccination induces potent neutralization in mice, rabbits, and cynomolgus macaques
• Vaccination protects macaques against a high-dose SARS-CoV-2 challenge

## Graphical Abstract

**Figure:**
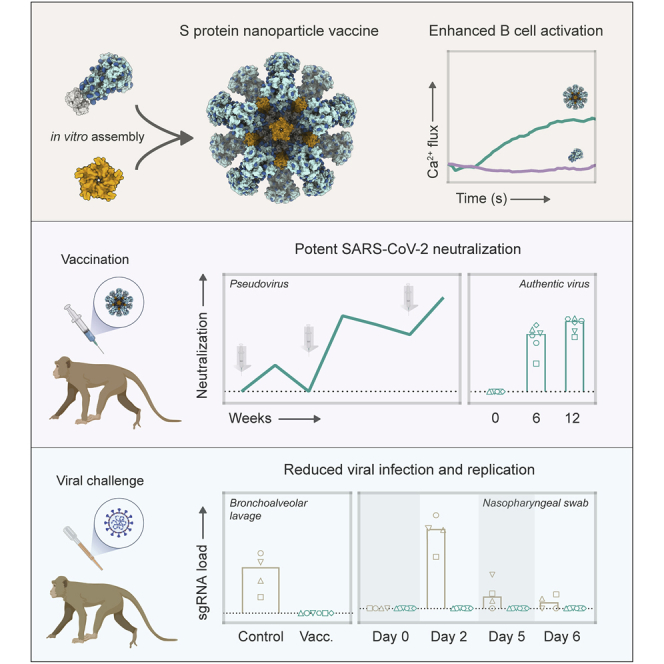

## Teaser

Brouwer et al. present preclinical evidence in support of a COVID-19 vaccine candidate, designed as a self-assembling two-component protein nanoparticle displaying multiple copies of the SARS-CoV-2 spike protein, which induces strong neutralizing antibody responses and protects from high-dose SARS-CoV-2 challenge.

## Introduction

Severe acute respiratory syndrome coronavirus 2 (SARS-CoV-2) has rapidly spread across the globe and infected more than 100 million individuals since late 2019 (https://covid19.who.int/). SARS-CoV-2 causes coronavirus disease 2019 (COVID-19), which manifests itself as a mild respiratory illness in most infected individuals but can lead to acute respiratory distress syndrome and death in a significant percentage of cases. As of February 1^st^, 2021, COVID-19 has caused over 2 million casualties and continues to place a significant burden on healthcare systems and economies worldwide. Hence, the development of a safe and effective vaccine that can prevent SARS-CoV-2 infection and transmission has rapidly become a top priority.

Recent studies suggest that SARS-CoV-2-specific neutralizing antibody (NAb) titers are an important immune correlate of protection (Addetia et al., 2020; Yu et al., 2020). This is supported by several passive immunization studies showing that administration of potent neutralizing SARS-CoV-2-specific monoclonal antibodies (mAbs) can significantly reduce lung viral loads (Cao et al., 2020; Rogers et al., 2020). Thus, a successful vaccine will likely need to induce a potent NAb response. The main target for NAbs on SARS-CoV-2 is the spike (S) protein. This homotrimeric glycoprotein is anchored in the viral membrane and consists of the S1 domain, which contains the receptor-binding domain (RBD) for the host cell receptor angiotensin-converting enzyme 2 (ACE2), and the S2 domain, which contains the fusion peptide. Upon binding to ACE2, the prefusion S protein undergoes several structural changes that induce a shift to a postfusion state that enables merging of the viral envelope and host cell membrane (Shang et al., 2020). As most NAb epitopes are presented on the prefusion conformation, vaccine candidates should include the prefusion S protein, which, as for other class I viral fusion proteins (Krarup et al., 2015; Sanders et al., 2002), can be stabilized by appropriately positioned proline substitutions (Pallesen et al., 2017; Walls et al., 2020a; Wrapp et al., 2020).

More than 200 candidate vaccines are currently under preclinical or clinical evaluation, and several have been licensed (https://www.who.int/publications/m/item/draft-landscape-of-covid-19-candidate-vaccines). Recombinant subunit vaccines provide a welcome alternative to the inactivated, viral-vector- and RNA-based vaccines that are currently in phase 3 clinical trials or in use, as they have a track record of safety and efficacy. In addition, recombinantly expressed S proteins represent an efficient antigen to induce potent NAb responses, as recently reported in non-human primate studies (Guebre-Xabier et al., 2020; Liang et al., 2020; Tian et al., 2021).

A well-established strategy to generate strong humoral immune responses against soluble antigens is multivalent antigen display (Bachmann and Jennings, 2010; Moyer et al., 2016). Nanoparticles presenting a high density of antigen on a repetitive array facilitate numerous immunological processes such as B cell activation, lymph node trafficking, and retention on follicular dendritic cells (Kelly et al., 2019; Tokatlian et al., 2019). Among the several nanoparticle designs that are currently being employed to present viral glycoproteins, self-assembling protein nanoparticle systems represent promising platforms, as they allow for efficient and scalable nanoparticle assembly (López-Sagaseta et al., 2015). Homomeric protein complexes such as the 24-subunit ferritin and 60-subunit mi3 nanoparticles self-assemble intracellularly and have been used to display immunogens such as influenza hemagglutinin (HA), HIV-1 Env, malaria cysteine-rich protective antigen (CyRPA), and also SARS-CoV-2 S protein and RBD (Bruun et al., 2018; He et al., 2020; Kanekiyo et al., 2013; Ma et al., 2020; Powell et al., 2021; Sliepen et al., 2015; Walls et al., 2020b). Recently, two-component 120-subunit icosahedral nanoparticles, such as the I53-50 and dn5 designs, have been developed that self-assemble *in vitro*, allowing for controlled nanoparticle formation. We and others have been using these nanoparticles to multivalently present trimeric type 1 viral fusion proteins of HIV-1, respiratory syncytial virus (RSV), and influenza (Boyoglu-Barnum et al., 2020; Brouwer et al., 2019; Marcandalli et al., 2019). Presentation of these immunogens on two-component protein nanoparticles significantly improved NAb titers, which supports pursuing this platform for the generation of nanoparticle immunogens displaying prefusion SARS-CoV-2 S proteins.

Here, we describe the generation and characterization of two-component protein nanoparticles displaying stabilized prefusion SARS-CoV-2 S proteins. Immunization studies in mice, rabbits, and macaques demonstrated that these nanoparticles induce robust humoral responses. Vaccinated macaques challenged with a high dose of SARS-CoV-2 virus had strongly reduced viral loads in both the upper and lower respiratory tracts and developed fewer lung lesions when compared to unvaccinated animals.

## Results

### SARS-CoV-2 S proteins can be displayed on two-component I53-50 nanoparticles

**Figure 1:**
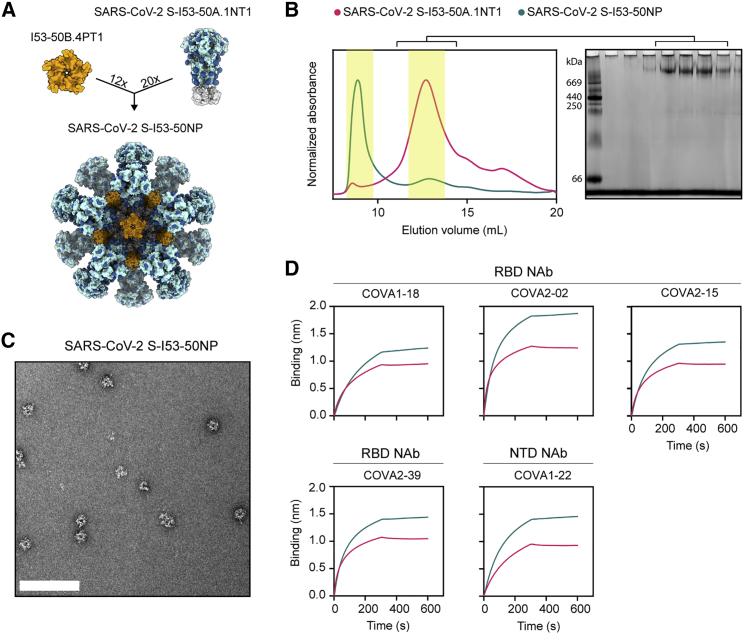
Biophysical and antigenic characterization of SARS-CoV-2 S-I53-50NPs (A) Schematic representation of 20 copies of SARS-CoV-2 S-I53-50A.1NT1 (SARS-CoV-2 S in light blue, glycans in dark blue, and I53-50A.1NT1 in white) and 12 copies of I53-50B.4PT1 assembling into SARS-CoV-2 S-I53-50NP. (B) Size exclusion chromatograms of SARS-CoV-2 S-I53-50A.1NT1 (magenta) and SARS-CoV-2 S-I53-50NP (green) run over a Superose 6 increase 10/300 GL column. The yellow columns specify the SEC fractions that were collected, pooled, and used. Blue native gel of pooled SARS-CoV-2 S-I53-50A.1NT1 SEC fractions. (C) Negative-stain electron microscopy (nsEM) analysis of assembled SARS-CoV-2 S-I53-50NPs. The white bar represents 200 nm. (D) BLI sensorgrams showing the binding of multiple SARS-CoV-2 NAbs to SARS-CoV-2 S-I53-50A.1NT1 (magenta) and SARS-CoV-2 S-I53-50NP (green). See also Figure S1.

The computationally designed I53-50 nanoparticle (I53-50NP) constitutes 20 trimeric (I53-50A or variants thereof) and 12 pentameric (I53-50B.4PT1) subunits that self-assemble to form monodisperse icosahedral particles with a diameter of ∼30 nm (Bale et al., 2016). To generate I53-50NPs presenting SARS-CoV-2 S proteins, we genetically fused the C terminus of the previously described stabilized prefusion S protein to the N terminus of the I53-50A variant, I53-50A.1NT1, using a glycine-serine linker (Figure 1A; Brouwer et al., 2020). The fusion protein was purified from transfected human embryonic kidney (HEK) 293F cell supernatant using nickel-nitrilotriacetic acid (Ni-NTA) purification followed by size exclusion chromatography (SEC). Collection of the appropriate SEC fractions yielded ∼2 mg/L trimeric SARS-CoV-2 S-I53-50A.1NT1 fusion protein (Figure 1B). To initiate assembly of nanoparticles presenting SARS-CoV-2 S proteins (SARS-CoV-2 S-I53-50NPs), the pooled trimer fractions were incubated overnight at 4°C with I53-50B.4PT1 in an equimolar ratio. The nanoparticle preparation was further purified using an additional SEC step to remove unassembled components. Negative-stain electron microscopy (nsEM) of the pooled higher-molecular-weight fractions revealed a considerable portion of monodisperse and well-ordered icosahedral nanoparticles (Figure 1C). Biolayer interferometry (BLI)-based binding experiments with a panel of SARS-CoV-2 S-protein-specific monoclonal NAbs, previously isolated from recovered COVID-19 patients (Brouwer et al., 2020), showed strong binding of RBD-specific COVA1-18, COVA2-02, COVA2-15, and COVA2-39 and N-terminal domain (NTD)-specific COVA1-22 to trimeric SARS-CoV-2 S-I53-50A.1NT1 and SARS-CoV-2 S-I53-50NP (Figure 1D). This suggests that presentation of SARS-CoV-2 S protein on I53-50NPs did not compromise the structure of these S protein epitopes. Altogether, SEC, nsEM, and BLI confirmed the successful generation of nanoparticles presenting multiple copies of the SARS-CoV-2 S protein.

**Figure S1:**
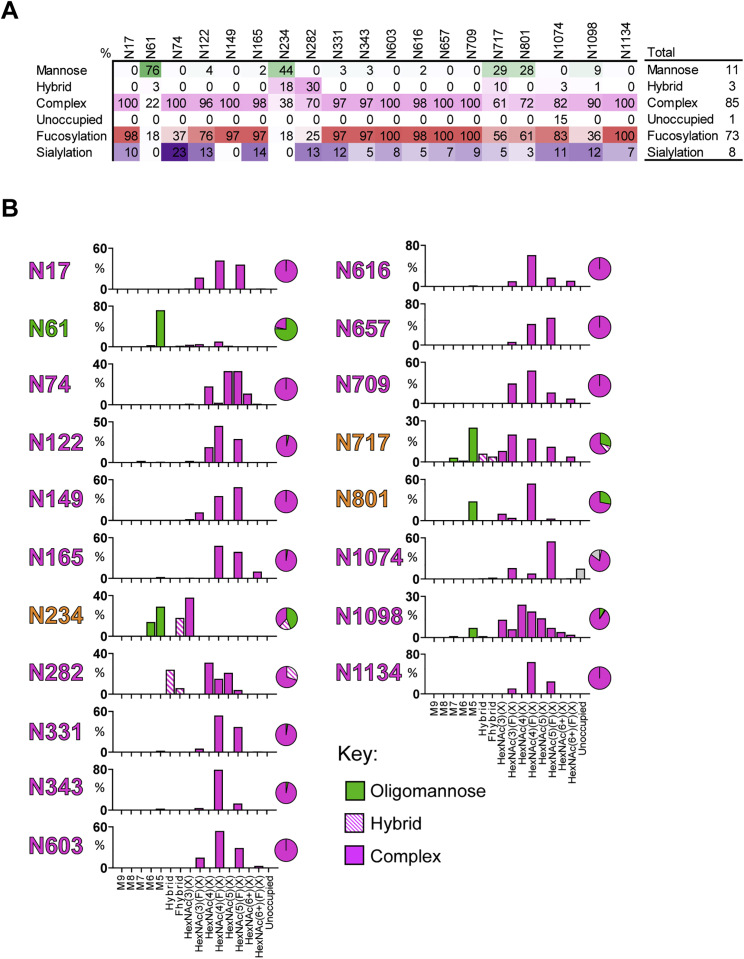
Site-specific glycan analysis of SARS-CoV-2 S I53-50NPs, related to Figure 1 (A) The table categorizes the glycan compositions into oligomannose-, hybrid-, and complex-type as well as the percentage of glycan species containing at least one fucose or one sialic acid residue. The overall averages are shown in the right-hand table. (B) Site-specific distribution of N-linked glycans. The graphs summarize quantitative mass spectrometric analysis of the glycan population present at individual N-linked glycosylation sites simplified into categories of glycans. The oligomannose-type glycan series (M9 to M5; Man_9_GlcNAc_2_ to Man_5_GlcNAc_2_) is colored green, afucosylated and fucosylated hybrid-type glycans (Hybrid & F Hybrid) dashed pink, and complex glycans grouped according to the number of antennae and presence of core fucosylation (HexNAc(3)(X) to HexNAc(6+)(F)(X)) and are colored pink. Unoccupancy of an N-linked glycan site is represented in gray. The pie charts summarize the quantification of these glycans. Glycan sites are colored according to oligomannose-type glycan content with the glycan sites labeled in green (80%−100% oligomannose), orange (30%−79% oligomannose) and pink (0%−29% oligomannose).

As approximately one-third of the mass of the SARS-CoV-2 S protein consists of N-linked glycans, we sought to identify the site-specific glycosylation of S protein presented on I53-50NPs using liquid chromatography-mass spectrometry (LC-MS). All sites presented high levels of occupancy, with only N1074 displaying 15% of sites lacking an N-linked glycan (Figure S1A). The compositions of glycans present at the 19 N-linked glycan sites on the S protein were determined and revealed a diverse range of structures (Figure S1B). Underprocessed oligomannose-type glycans were observed at sites N61, N234, N717, and N801 and to a lesser extent at N1098. The average oligomannose-type glycan content across all sites was 11%. Processed complex-type glycans were observed at all sites and were highly fucosylated (73%) but mostly lacked sialylation (8%) (Figures S1A and S1B). The glycoforms present on SARS-CoV-2 S-I53-50NPs are more processed compared to other recombinant S protein immunogens (Watanabe et al., 2020) but are reminiscent of the composition on S proteins presented on virions (Yao et al., 2020).

### SARS-CoV-2 S-I53-50NPs enhance activation of SARS-CoV-2 S-protein-specific B cells *in vitro*

**Figure 2:**
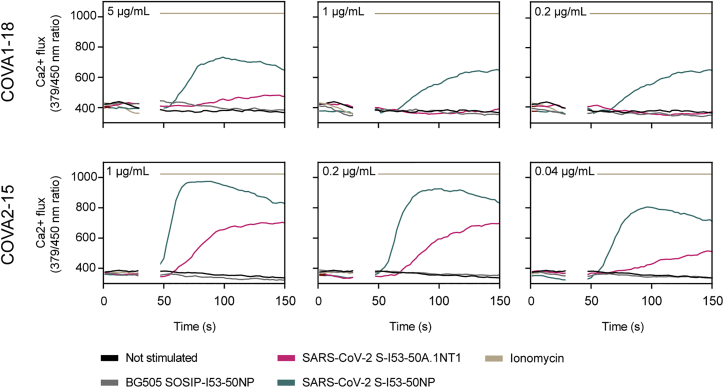
*In vitro* B cell activation by SARS-CoV-2 S-I53-50A.1NT1 and SARS-CoV-2 S-I53-50NPs B cells expressing the SARS-CoV-2 S-protein-specific NAbs COVA1-18 (top) or COVA2-15 (bottom) as BCRs were incubated with either SARS-CoV-2 S-I53-50A.1NT1 (magenta), SARS-CoV-2 S-I53-50NP (green), ionomycin (beige), or BG505 I53-50NP (gray) or not stimulated (black). The experiments were performed with 5, 1, 0.2, or 0.04 μg/mL SARS-CoV-2 S-I53-50A.1NT1, as indicated in the top left corner of each graph, or the equimolar amount of SARS-CoV-2 S or BG505 SOSIP on I53-50NPs. Ionomycin was used at 1 mg/mL as a positive control.

Multivalent display of antigens can enhance cognate B cell activation over soluble antigen (Antanasijevic et al., 2020; Brouwer et al., 2019; Veneziano et al., 2020). To assess if the same would apply for SARS-CoV-2 S-I53-50NPs, we generated B cells that expressed B cell receptors (BCRs) for previously described RBD-targeting monoclonal NAbs and measured their activation by monitoring Ca^2+^ influx *in vitro* (Brouwer et al., 2020). Soluble trimers only weakly activated COVA1-18-expressing B cells at the highest concentration used (5 μg/mL SARS-CoV-2 S-I53-50A.1NT1), while an equimolar amount of SARS-CoV-2 S presented on I53-50NPs efficiently activated the same B cells (Figure 2). COVA2-15-expressing B cells were activated by soluble SARS-CoV-2 S-I53-50A.1NT1 trimers but markedly more efficiently so by SARS-CoV-2 S-I53-50NP. The more efficient activation of COVA2-15-expressing B cells compared to those expressing COVA1-18 may be explained by the fact that COVA2-15 can interact with the RBD in both the up and down state, which may result in higher-avidity interactions (Brouwer et al., 2020). In control experiments, I53-50NPs displaying soluble HIV-1 envelope glycoproteins (BG505 SOSIP) did not activate any of the B cell lines. We conclude that SARS-CoV-2 S-I53-50NPs improve the activation of SARS-CoV-2-specific B cells compared to soluble trimers.

### SARS-CoV-2 S-I53-50NP vaccination induces robust NAb responses in small-animal models

**Figure 3:**
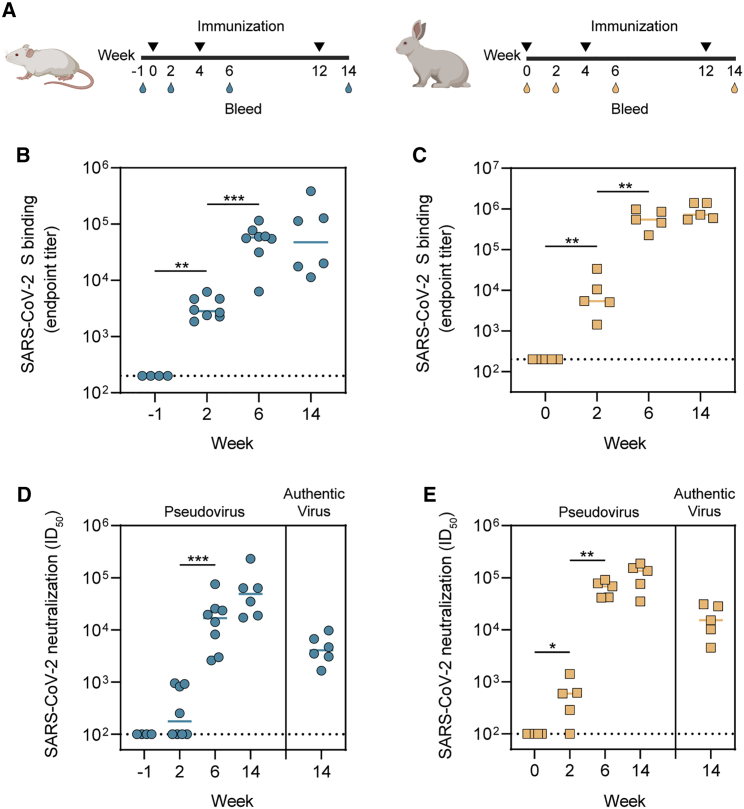
Immunogenicity of SARS-CoV-2 S-I53-50NPs in mice and rabbits (A) Study schedule in mice (left) and rabbits (right). Black triangles indicate the time points of immunization and drops indicate the bleeds. (B) ELISA endpoint titers for SARS-CoV-2 S-protein-specific IgG in mice. (C) ELISA endpoint titers for SARS-CoV-2 S-protein-specific IgG in rabbits. (D) SARS-CoV-2 pseudovirus and authentic virus neutralization titers in mice. (E) SARS-CoV-2 pseudovirus and authentic virus neutralization in rabbits. In (B) and (C), due to limited volumes of sera at week −1, random pairs of mice sera were pooled. At week 6, two animals were sacrificed. In (B)–(E), the median titers are indicated by a bar. Titers between boosts were compared using the Mann-Whitney U test (^∗^p < 0.05; ^∗∗^p < 0.01; ^∗∗∗^p < 0.001).

We assessed the immunogenicity of SARS-CoV-2 S-I53-50NPs in two small-animal models. Eight BALB/c mice were immunized with 10 μg SARS-CoV-2 S presented on I53-50NPs, adjuvanted in polyinosinic-polycytidylic acid (poly(I:C)). In addition, five New Zealand white rabbits were immunized with 30 μg SARS-CoV-2 S presented on I53-50NPs, adjuvanted in squalene emulsion. Mice and rabbits received three subcutaneous and intramuscular immunizations, respectively, at weeks 0, 4, and 12 (Figure 3A). The adjuvants were chosen based on our previous positive experiences with them in the respective animal models.

Two weeks after the first immunization, mice and rabbits induced detectable SARS-CoV-2 S-protein-specific immunoglobulin G (IgG) titers, as determined by an enzyme-linked immunosorbent assay (ELISA), with a median endpoint binding titer of 2,920 and 5,105 respectively. In mice, median endpoint titers were further boosted after the second immunization to 57,943 at week 6 and slightly decreased after the third immunization to 47,643 at week 14 (Figure 3B). In rabbits, median endpoint titers were boosted to 544,503 at week 6 and 594,292 at week 14 (Figure 3C). Neutralization of SARS-CoV-2 pseudovirus was already detectable in the majority of mice and rabbit sera 2 weeks after the first immunization. NAb titers, which are represented by the inhibitory dilutions at which 50% neutralization is attained (ID_50_ values), increased to a median of 16,792 at week 6 and 49,039 at week 14 in mice (Figure 3D; Table S1). In rabbits, NAb titers were boosted to a median ID_50_ of 68,298 and 135,128 at weeks 6 and 14, respectively (Figure 3E; Table S1). Neutralization titers of authentic SARS-CoV-2 virus at week 14 reached a median ID_50_ of 4,065 and 15,110 in mice and rabbits, respectively (Figures 3D and 3E; Table S1). Collectively, we showed that SARS-CoV-2 S-I53-50NPs were able to induce robust binding and NAb responses in both mice and rabbits after two and three immunizations.

### SARS-CoV-2 S-I53-50NP vaccination induces potent humoral responses in cynomolgus macaques

**Figure 4:**
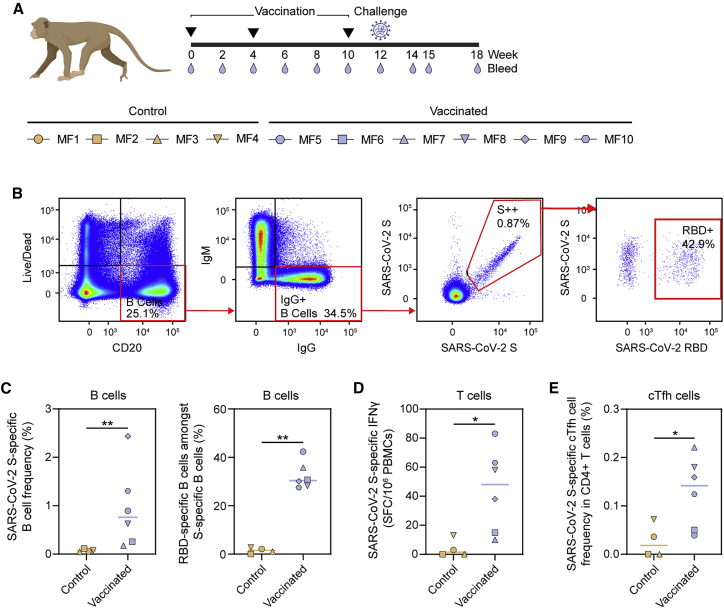
SARS-CoV-2 S-protein-specific B and T cell responses induced by SARS-CoV-2 S-I53-50NPs in cynomolgus macaques (A) Vaccination, challenge, and sampling schedule in cynomolgus macaques. Black triangles indicate the time points of vaccination and drops mark the bleeds. The symbols identifying individual macaques are used consistently throughout Figures 4, 5, and 6. (B) Representative gating strategy, depicting the analysis of SARS-CoV-2 S protein and RBD-specific IgG^+^ B cells, shown for one vaccinated macaque. The lymphocyte population was selected, and doublets were excluded (not shown). Gating strategy from the left to the right: viable B cells (live/dead^−^CD20^+^), IgG^+^ B cells (IgM^−^IgG^+^), SARS-CoV-2 S protein (double probe staining), and RBD-specific (single probe staining) IgG^+^ B cells. (C) SARS-CoV-2 S-protein-specific B cell frequencies within the IgG^+^ population in control and vaccinated macaques (left). Percentages of SARS-CoV-2 RBD-specific B cells within the population of SARS-CoV-2 S-protein-specific IgG^+^ B cells (right). (D) Number of interferon-γ (IFNγ)-secreting cells after *ex vivo* stimulation with SARS-CoV-2 S protein as analyzed by ELISpot and plotted as spot-forming cells (SFCs) per 1.0 × 10^6^ peripheral blood mononuclear cells (PBMCs). (E) Frequency of SARS-CoV-2 S-protein-specific Tfh cells (CD69^+^CD154^+^CXCR5^+^) in the total CD4^+^ T cell population. PBMCs were stimulated overnight with SARS-CoV-2 S protein and Tfh activation was assessed the next day by analyzing CD69 and CD154 expression by flow cytometry. The gating strategy used to identify this population is shown in Figure S3. In (C)-(E) medians are indicated by a bar. Groups were compared using the Mann-Whitney U test (^∗^p < 0.05; ^∗∗^p < 0.01). See also Figures S2 and S3.

The high binding and neutralization titers in mice and rabbits supported subsequent assessment of the immunogenicity of SARS-CoV-2 S-I53-50NPs in cynomolgus macaques, an animal model that is immunologically closer to humans. Six cynomolgus macaques were immunized with 50 μg SARS-CoV-2 S-I53-50NPs formulated in monophospholipid A (MPLA) liposomes by the intramuscular route at weeks 0, 4, and 10 (Figure 4A). The MPLA liposome adjuvant was chosen because it is used in several human clinical trials (e.g., NCT03816137, NCT03961438, and NCT04046978) and is a component of the widely used AS01 adjuvant, which was safe and effective in several phase 3 clinical trials (Lal et al., 2018; Agnandji et al., 2012).

**Figure S2:**
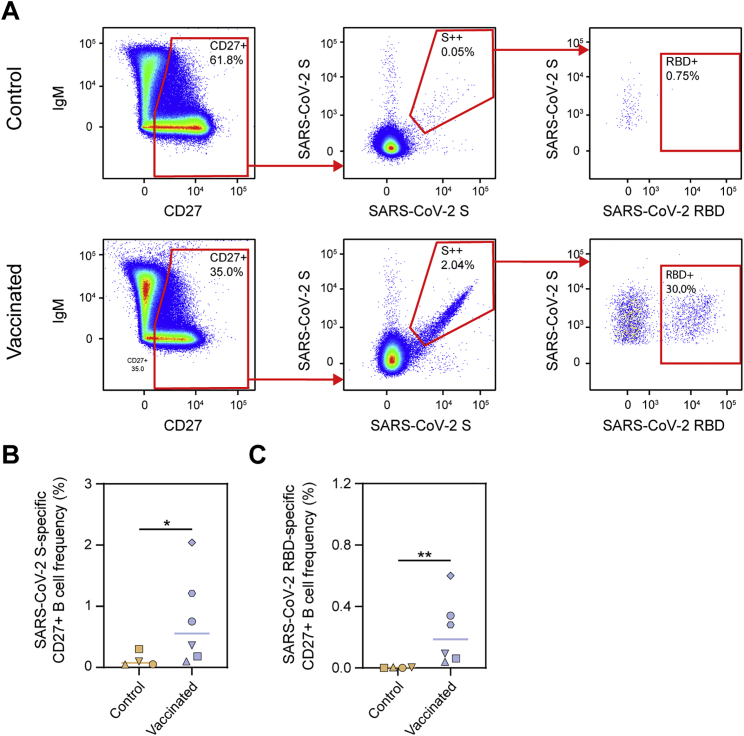
SARS-CoV-2 S-protein-specific CD27^+^ B cell responses in control and vaccinated macaques, related to Figure 4 (A) Representative gating strategy for the identification of SARS-CoV-2 S protein and RBD-specific CD27+ B cells for control (top) and vaccinated (bottom) macaques. The live B cell population was selected and doublets were excluded (not shown). (B) Frequency of SARS-CoV-2 S protein-specific cells among CD27+ B cells in control and vaccinated macaques. (C) Frequency of SARS-CoV-2 RBD-specific cells among CD27+ B cells in control and vaccinated macaques. In (B) and (C) bars indicate median. Groups were compared using the Mann-Whitney U test (^∗^p < 0.05; ^∗∗^p < 0.01).

**Figure S3:**
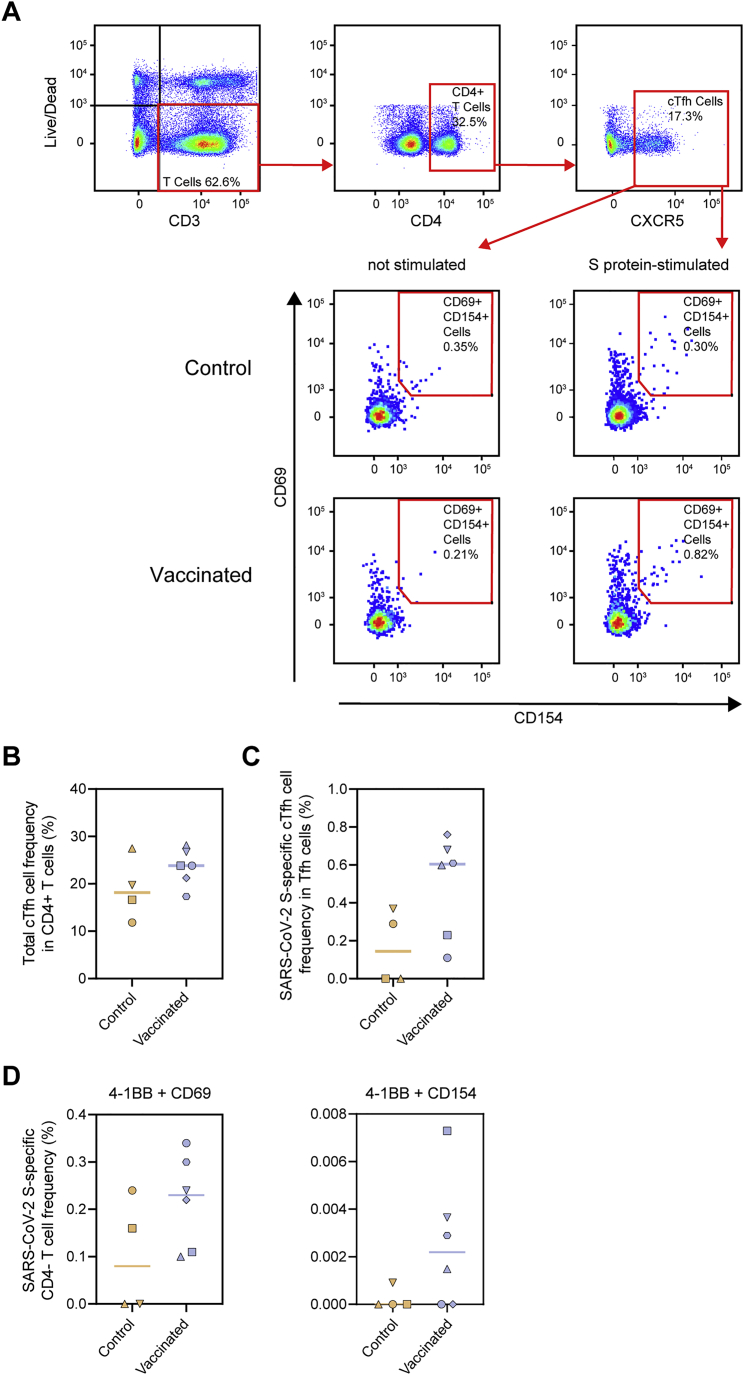
SARS-CoV-2 S-protein-specific Tfh cell and CD4^−^ T cell responses in control and vaccinated macaques, related to Figure 4 (A) Representative gating strategy for the identification of SARS-CoV-2 S protein-specific Tfh cells. PBMCs were stimulated overnight with SARS-CoV-2 S protein and Tfh activation was assessed the next day by analyzing CD69 and CD154 expression. (B) Frequency of total Tfh cells in CD4+ T cell population following stimulation with SARS-CoV-2 S protein. (C) Frequency of SARS-CoV-2 S-protein specific Tfh cells within the total Tfh cell population. Corresponding background (i.e., frequency of activated Tfh cells in non-stimulated cells) has been subtracted from each data point. (D) Frequency of SARS-CoV-2 S protein-specific cells among CD4- T cells (CD8+ T cells by inference) as determined by the AIM assay using 4-1BB + CD69 (left) and 4-1BB + CD154 (right) as activation markers. Similar to (A), CD4- cells were stimulated overnight with SARS-CoV-2 S protein and activation was assessed using activation markers. Corresponding background (i.e., frequency of activated CD4- cells in non-stimulated cells) has been subtracted from each data point. In (B)-(D) bars indicate median.

To analyze the frequency of S protein and RBD-specific IgG-positive B cells in macaques after vaccination, peripheral blood mononuclear cells at week 12 were gated on the expression of CD20 and IgG and stained with fluorescently labeled prefusion SARS-CoV-2 S protein or RBD (Figure 4B). We observed a clear expansion of SARS-CoV-2 S-protein-specific B cells by vaccination, which constituted on average ∼1% of total B cells (Figure 4C). Within the population of IgG-positive SARS-CoV-2 S-protein-specific B cells, on average, ∼30% bound to the RBD (Figure 4C). Within the CD27^+^ B cell population (marker for memory B cells), on average, 0.77% were SARS-CoV-2 S protein specific, of which, again, ∼30% were specific for the RBD (Figure S2). In addition to B cells, SARS-CoV-2 S-protein-specific T cells were also markedly expanded, as determined by an enzyme-linked immune absorbent spot assay (ELISpot) (Figure 4D). Furthermore, we studied circulating T follicular helper cells (cTfh cells), the circulating counterpart of germinal center Tfh cells (Heit et al., 2017; Vella et al., 2019). We observed pronounced expansion of S-protein-specific cTfh cells (CD69^+^ CD154^+^ CXCR5^+^) within the CD4^+^ T cell subset, as determined by the activation-induced marker (AIM) assay (Figures 4E and S3A–S3C). Within CD4^−^ T cells (CD8^+^ T cells by inference), a trend toward higher expansion of cells was observed in the vaccinated macaques (Figure S3D)

**Figure 5:**
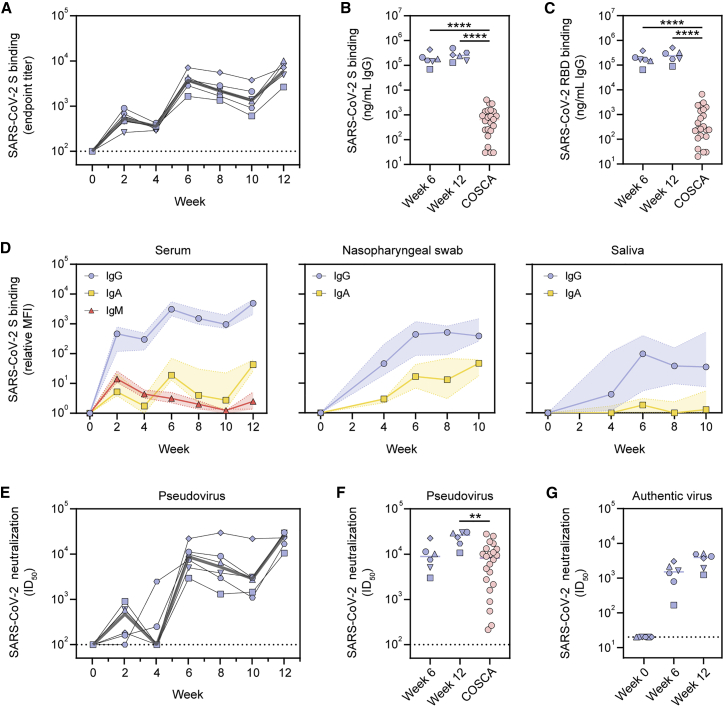
Serological responses induced by SARS-CoV-2 S-I53-50NPs in cynomolgus macaques (A) ELISA endpoint titers for SARS-CoV-2 S-protein-specific IgG. The gray line represents the median titers over time. (B) SARS-CoV-2 S-protein-specific binding titers at weeks 6 and 12 in macaques compared to those in convalescent humans from the COSCA cohort. Patient samples were taken 4 weeks after onset of symptoms. (C) SARS-CoV-2 RBD-specific binding titers at weeks 6 and 12 in macaques compared to those in convalescent humans from the COSCA cohort. (D) Relative mean fluorescence intensity (MFI) of IgG, IgA, and IgM binding to SARS-CoV-2 S protein measured with a Luminex-based serology assay in serum samples, nasopharyngeal swabs, and saliva samples. Shown are medians with the shaded areas indicating the interquartile ranges. (E) SARS-CoV-2 pseudovirus neutralization titers. The gray line represents median titers. (F) SARS-CoV-2 pseudovirus neutralization titers at weeks 6 and 12 in macaques compared to those in convalescent humans from the COSCA cohort. (G) SARS-CoV-2 authentic virus neutralization titers at weeks 6 and 12. The bars show the median titers. In (B), (C), and (F), groups were compared using the Mann-Whitney U test (^∗∗^p < 0.01; ^∗∗∗∗^p < 0.0001). The bars indicate median titers. The dotted lines indicate the lowest serum dilution. See also Figure S4.

Sera of the immunized macaques exhibited SARS-CoV-2 S-protein-specific binding IgG responses with median endpoint titers of 211, 1,601 and 2,190, at weeks 2, 6, and 12, respectively (Figure 5A). To compare binding titers to SARS-CoV-2 S protein and RBD between sera from vaccinated macaques and those from convalescent humans from the COVID-19 Specific Antibodies (COSCA) cohort (sampled ∼4 weeks after the onset of symptoms) (Brouwer et al., 2020), a different ELISA protocol was used. Specifically, binding responses were compared to a standard curve of species-specific polyclonal IgG so that a semiquantitative measure of specific IgG concentrations could be obtained. Week 6 and 12 sera elicited markedly higher IgG binding titers to SARS-CoV-2 S protein than serum from convalescent humans (Figure 5B). This was also the case for RBD-specific binding titers (Figure 5C).

**Figure S4:**
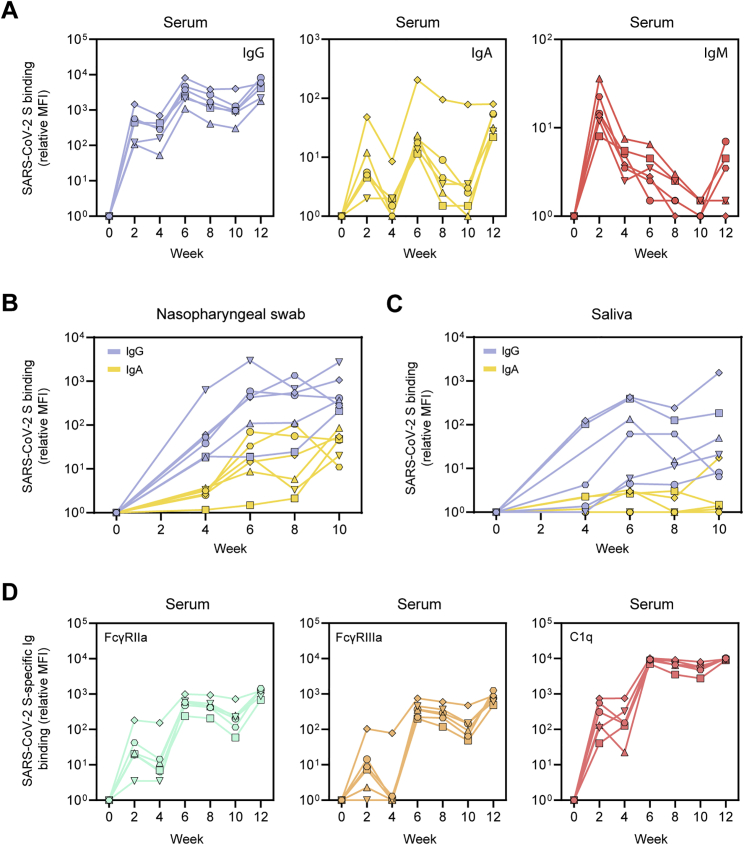
SARS-CoV-2 S-protein-specific Ig levels and Fc-receptor binding in vaccinated cynomolgus macaques in samples from diverse anatomical sites, related to Figure 5 (A) Relative MFI of IgG (left), IgA (middle) and IgM (right) binding to SARS-CoV-2 S protein measured with a Luminex-based serology assay in serum samples. (B) Relative MFI of IgG and IgA binding to SARS-CoV-2 S in nasopharyngeal swabs. (C) Relative MFI of IgG and IgA binding to SARS-CoV-2 S in saliva. (D) Relative MFI of FcγRIIa (left), FcγRIIIa (middle) and C1q (right) binding to SARS-CoV-2 S protein-specific IgG in serum samples.

Using a custom Luminex-bead-based serological assay, we analyzed the induction of several Ig isotypes in serum, nasopharyngeal swab, and saliva samples of the vaccinated macaques over time. S-protein-specific IgG levels in serum showed a similar course as observed by ELISA (Figures 5D and S4A). This was also the case for IgA, albeit with a more rapid onset and waning after vaccination. As expected, IgM levels peaked after the first immunization and gradually waned thereafter, presumably as a result of Ig class switching (Figures 5D and S4A). We also observed an increase in S-specific IgG and IgA levels in nasopharyngeal swabs and saliva after consecutive immunizations. This was particularly clear for IgG in nasopharyngeal swabs (Figures 5D and S4B). The results in saliva samples were more variable (Figures 5D and S4C). Thus, in addition to a systemic antibody response, SARS-CoV-2 S-I53-50NPs induced detectable mucosal IgA and IgG responses, a relevant finding considering that the respiratory mucosa is the first port of entry for SARS-CoV-2 (Sungnak et al., 2020). Finally, we analyzed the ability of SARS-CoV-2 S-protein-specific serum antibodies to bind to immune proteins that play a role in Fc-mediated effector functions. The levels of FcγRIIa, FcγRIIIa, and C1q binding tracked with IgG levels, suggesting that the induced IgGs can perform effector functions such as antibody-dependent cellular-cytotoxicity, phagocytosis, and complement activation (Figure S4D).

Serum neutralization titers in the vaccinated macaques were overall robust. Two weeks after the first immunization, macaques induced pseudovirus neutralization with a median ID_50_ of 475. The second immunization increased the neutralization titers to a median ID_50_ of 8,865, which then declined only modestly up to the third immunization. NAb titers were further increased to a median ID_50_ of 26,361 at week 12 (Figure 5E; Table S2). At week 6, the neutralization titers were similar to those in sera from convalescent humans, but neutralization titers at week 12 were significantly higher in vaccinated macaques than convalescent humans (median ID_50_ of 26,361 versus 8,226, p = 0.0012) (Figure 5F). Neutralization of authentic SARS-CoV-2 was lower than that of pseudovirus but remained potent (median ID_50_ of 1,501 and 3,942 at weeks 6 and 12, respectively) (Figure 5G; Table S2).

### SARS-CoV-2 S-I53-50NP vaccination protects cynomolgus macaques from high-dose SARS-CoV-2 challenge

**Figure 6:**
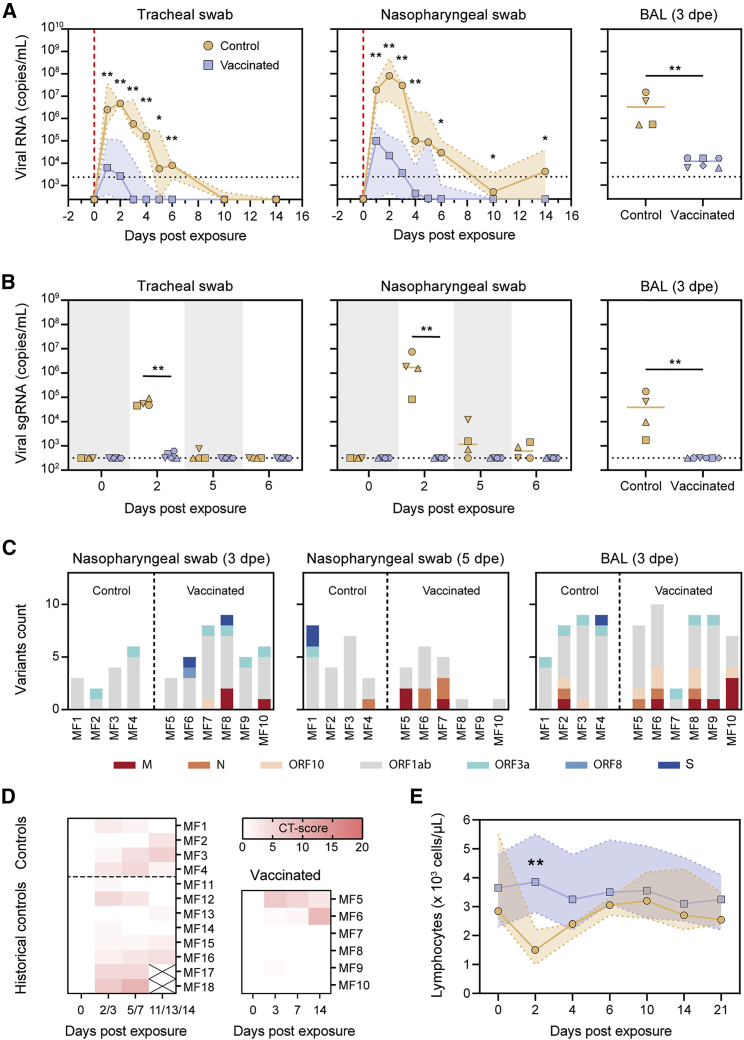
Protective efficacy of SARS-CoV-2 S-I53-50NPs in cynomolgus macaques (A) Median RNA viral loads in tracheal swabs (left) and nasopharyngeal swabs (middle) of control and vaccinated macaques after challenge. The shaded area indicates the range. Viral loads in control and vaccinated macaques after challenge in BAL are shown (right). Bars indicate median viral loads. Vertical red dotted lines indicate the day of challenge. Horizontal dotted lines indicate the limit of quantification. (B) sgRNA viral loads in tracheal swabs (left), nasopharyngeal swabs (middle), and BAL (right) of control and vaccinated macaques after challenge. Bars indicate median viral loads. Dotted line indicates the limit of detection. (C) Emerged viral variants found by viral sequencing in nasopharyngeal swabs at 3 dpe (left) and 5 dpe (middle) and BAL at 3 dpe. Colors indicate the open reading frames (ORFs) in which mutations were found, as depicted in the legend below. For a list of all identified variants, see Table S3. Note that the challenge stock already contained two viral variants, V367F in S protein and G251V in ORF3a. (D) Lung CT scores of control and vaccinated macaques over the course of 14 dpe. CT score includes lesion type (scored from 0 to 3) and lesion volume (scored from 0 to 4) summed for each lobe. (E) Median lymphocyte counts over time in the blood of control and vaccinated macaques after challenge. Shaded area indicates the range. Symbols are the same as indicated in the left panel in (A). In (A), (B), and (E), groups were compared using the Mann-Whitney U test (^∗^p < 0.05; ^∗∗^p < 0.01). See also Figures S5 and S6.

**Figure S5:**
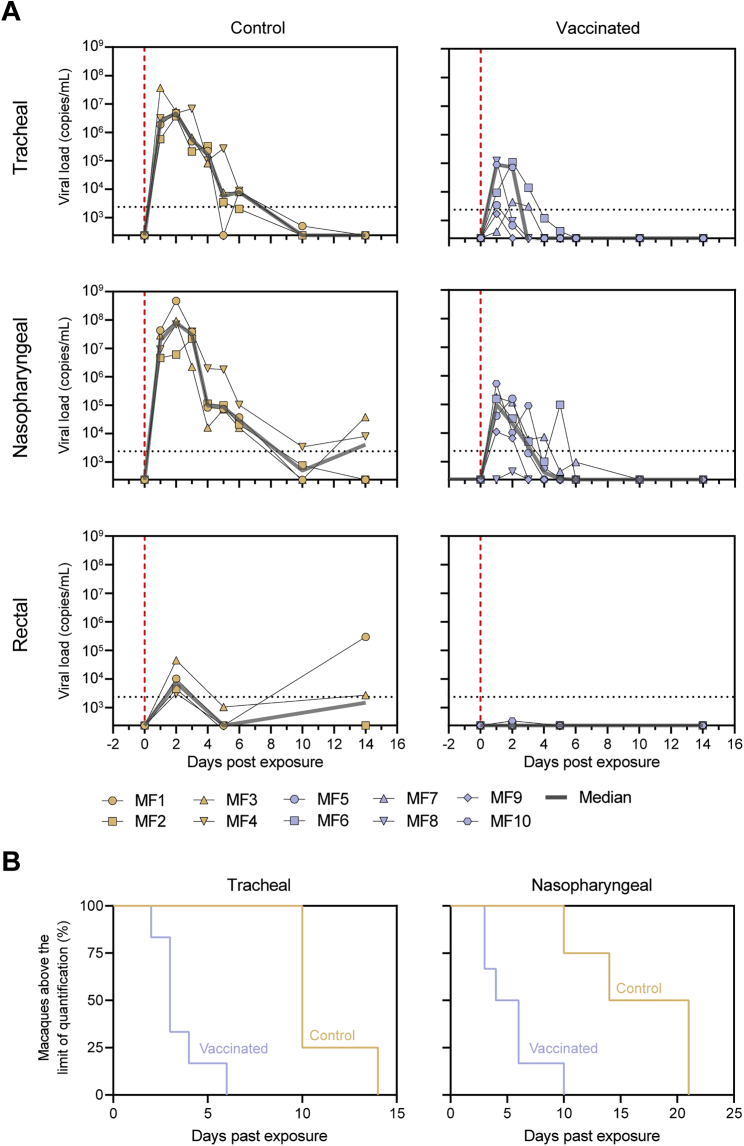
Viral loads in control and vaccinated cynomolgus macaques after SARS-CoV-2 challenge, related to Figure 6 (A) RNA viral loads in tracheal swabs (top), nasopharyngeal swabs (middle) and rectal samples (bottom) of control (left) and SARS-CoV-2 S-I53-50NP vaccinated macaques (right) after challenge. The gray line represents the median viral load. Vertical red dotted lines indicate the day of challenge. Horizontal dotted lines indicate the limit of quantification. Symbols identify individual macaques. (B) Percentage of macaques in which the RNA viral loads is above the limit of quantification over time in tracheal swabs (left) and nasopharyngeal swabs (right).

To assess the protective potential of SARS-CoV-2 S-I53-50NPs, vaccinated macaques and contemporaneous control macaques (n = 4) were infected with a total dose of 1 × 10^6^ plaque-forming units (PFUs) of a primary SARS-CoV-2 isolate (BetaCoV/France/IDF/0372/2020; passaged twice in VeroE6 cells) by combined intranasal (0.25 mL in each nostril) and intratracheal (4.5 mL in the trachea) routes at week 12, 2 weeks after the final immunization. This represents a high-dose challenge in comparison with other studies, where 10- to 100-fold lower doses were used (van Doremalen et al., 2020; Feng et al., 2020; Guebre-Xabier et al., 2020; Mercado et al., 2020; Patel et al., 2020; Yu et al., 2020). Control animals had high viral load levels in nasopharyngeal and tracheal samples (swabs), as assessed by qRT-PCR for viral RNA, as early as 1 day post-exposure (dpe). In tracheal samples, viral loads peaked between 1 and 3 dpe, with a median value of 6.9 log_10_ copies/mL. Subsequently, viral loads progressively decreased, and all animals had undetectable viral loads by 14 dpe (Figures 6A and S5). Similar profiles were observed in nasopharyngeal swabs, although viral loads remained detectable in some macaques at 14 dpe (Figures 6A and S5). Viral loads were markedly lower in rectal samples but stayed above the limit of detection during the course of sampling for two out of four control macaques (Figure S5). Viral subgenomic RNA (sgRNA), which is believed to reflect viral replication, peaked at 2 dpe in tracheal and nasopharyngeal swabs (median viral sgRNA load 4.8 and 6.1 log_10_,respectively) and was still detectable (>2.5 log_10_ copies/mL of viral sgRNA) at 5 and 6 dpe in the nasopharynx for three and two animals, respectively (Figure 6B).

In vaccinated macaques, median peak viral loads were 300-fold lower in tracheal samples (6.8 log_10_ versus 4.3 log_10_; p = 0.0095) and 500-fold lower in nasopharyngeal samples compared to unvaccinated controls (7.9 log_10_ versus 5.2 log_10_; p = 0.0095). With the exception of MF7, all vaccinated animals had undetectable loads at 6 dpe in tracheal and nasopharyngeal swabs (Figures 6A and S5). In addition to the upper airways, SARS-CoV-2 S-I53-50NP vaccination significantly decreased viral loads in the lower airways, as demonstrated by a 275-fold lower median viral load in the bronchoalveolar lavage (BAL) (6.5 log_10_ versus 4.1 log_10_; p = 0.0095). Viral replication was also significantly reduced in vaccinated animals. Only two out of six vaccinated animals (MF5 and MF6) showed detectable sgRNA in the trachea at 2 dpe, and median viral loads were 160-fold lower than in control animals (4.7 log_10_ versus 2.5 log_10_; p = 0.0095). In the nasopharynx, sgRNA remained below the limit of detection at any of the time points. At 2 dpe, median sgRNA loads in the vaccinated animals were 5,400-fold lower than in controls (6.2 log_10_ versus 2.5 log_10_; p = 0.0048) (Figure 6B). In BAL samples, we observed a 120-fold reduction of median sgRNA at day 3 dpe (4.6 log_10_ versus 2.5 log_10_; p = 0.0048) (Figures 6A and 6B).

**Figure S6:**
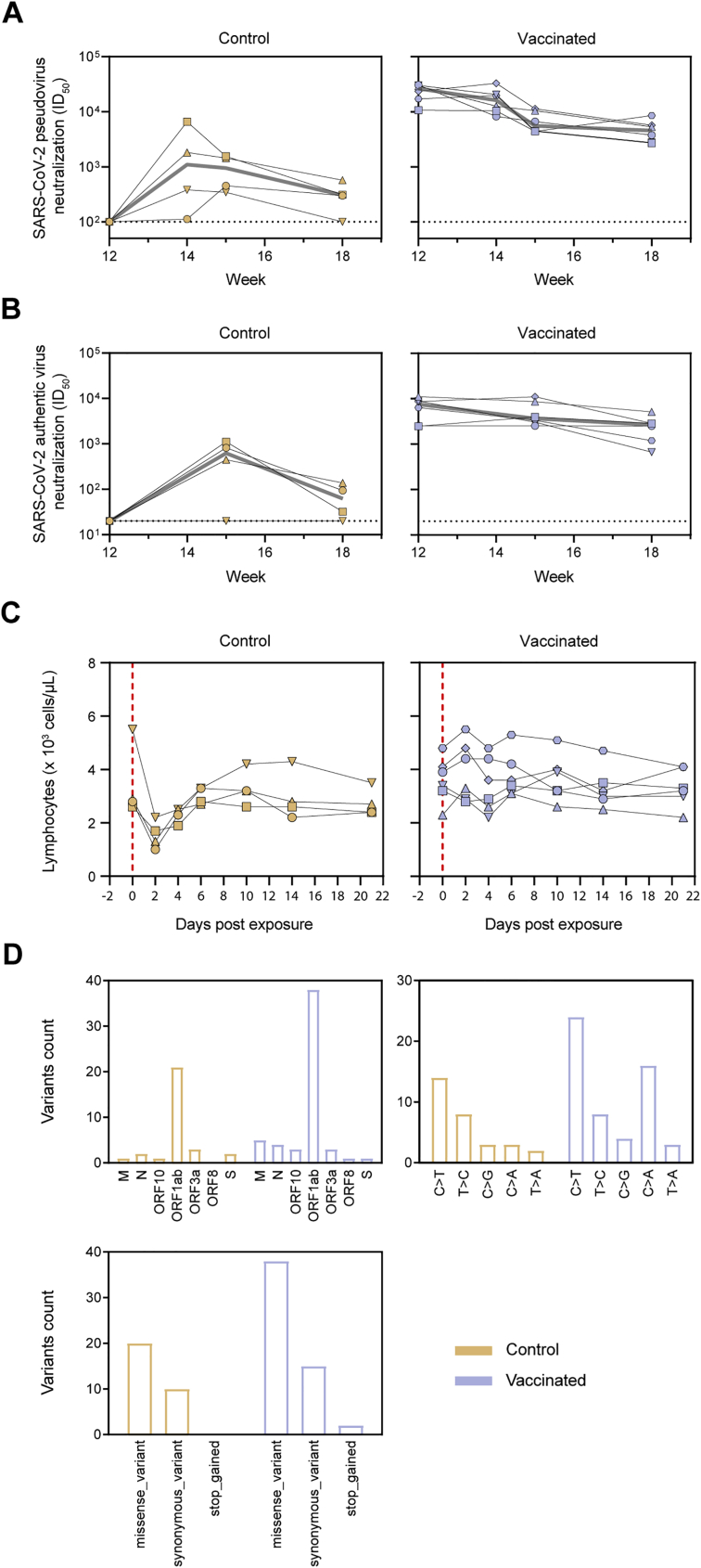
Anamnestic immune response, lymphocyte counts, and emerged viral sequence variants in control and vaccinated cynomolgus macaques after SARS-CoV-2 challenge, related to Figure 6 (A) SARS-CoV-2 pseudovirus neutralization titers. The gray line represents the median titers over-time. (B) SARS-CoV-2 authentic virus neutralization titers. The gray line represents the median titers over-time. (C) Lymphocyte counts over time in the blood of control and SARS-CoV-2 S-I53-50NP vaccinated macaques after challenge. Vertical red dotted lines indicate the day of challenge. In (A)-(C) Symbols identify individual macaques as indicated Figure S5A. (D) Sum of viral variants found by viral sequencing, in nasopharyngeal swabs at 3 dpe and 5 dpe, and in BAL at 3 dpe, specified for the ORF in which it was found (top left) the type of nucleotide change (top right) and the effect is has on the amino acid sequence (bottom; missense_variant = amino acid change, synonymous_variant = no amino acid change, stop_gained = introduction of a stop codon). For a list of all identified variants see Table S3.

An anamnestic response after challenge (i.e., an increase in NAb titers following challenge after vaccination) implies that vaccination is unable to induce sterilizing immunity. We observed no increase in median NAb titers in vaccinated macaques at 2, 3, and 6 weeks after challenge, in contrast to the control animals (Figure S6). Instead, NAb titers generally continued to wane, suggesting that vaccine-induced immunity rapidly controlled infection following challenge, preventing a boost of the immune system.

To assess the potential emergence of viral escape mutants in macaques after challenge, viral RNA in the challenge inoculum, nasal swabs at 3 and 5 dpe, and in BAL at 3 dpe was sequenced. Two main viral variants were identified in the inoculum (V367F in the S protein and G251V in ORF3a), which were later also found in the nasopharyngeal swabs and BAL samples. A median of six subclonal mutations were found per sample over all corresponding time points and anatomical sites, but no major differences were observed between control and vaccinated animals (Figure 6C). The majority of the variants observed in ORF1ab were mostly missense mutations and consisted of a C > T nucleotide change (Figure S6D; Table S3). A distinct variant in the S sequence arose in two vaccinated macaques at day 3 in the nasopharyngeal swab but disappeared at day 5 post-challenge, suggesting that no NAb escape mutations emerged in vaccinated animals (Figure 6C).

### Vaccinated cynomolgus macaques have reduced clinical manifestations

Similar to previous observations (Maisonnasse et al., 2020; Yu et al., 2020), during the first 14 dpe, all contemporaneous and historical control animals showed mild pulmonary lesions characterized by nonextended ground-glass opacities (GGOs) detected by chest computed tomography (CT) (Figure 6D). By contrast, only three out of six vaccinated animals showed low CT scores characteristic of mild and nonextended GGOs. Of note, the vaccinated macaque (MF6) with the highest CT score at day 14 showed the lowest S protein and RBD binding titers, the lowest NAb titers, and the highest viral load and sgRNA at day 5 pe. Whereas all control animals experienced lymphopenia at 2 dpe, corresponding probably to the installation of the response to infection, lymphocyte counts remained stable after challenge for the vaccinated macaques (Figures 6E and S6), in agreement with the absence of detectable anamnestic response. Together, these data further support that vaccination with SARS-CoV-2 S-I53-50NPs reduces the severity of infection.

## Discussion

The development and distribution of a protective vaccine is paramount to bring the SARS-CoV-2 pandemic to a halt. Over the last few months, numerous vaccine candidates of different modalities have entered clinical and preclinical studies, including inactivated-virus-, DNA-, mRNA-, vector-, and protein-based vaccines (Klasse et al., 2020). Multivalent presentation of RSV and influenza antigens on two-component self-assembling protein nanoparticles has generated remarkably potent immune responses in non-human primates (Boyoglu-Barnum et al., 2020; Marcandalli et al., 2019). Recently, I53-50NPs presenting the RBD of SARS-CoV-2 induced potent NAb titers and significantly decreased viral loads in humanized mice (Walls et al., 2020b). This RBD-based SARS-CoV-2 vaccine, as well as an RSV vaccine using the I53-50 nanoparticle platform, is in clinical development (https://www.icosavax.com/), illustrating the feasibility of large-scale good manufacturing practices (GMP) production. Here, we show that I53-50NPs presenting 20 copies of prefusion SARS-CoV-2 S protein induce robust NAb responses in mice, rabbits, and cynomolgus macaques. Vaccination of the latter prevented lymphopenia, reduced lung damage, and significantly reduced viral loads and replication in both the upper and lower respiratory tract, suggesting that SARS-CoV-2 S-I53-50NPs could reduce the risk of severe SARS-CoV-2-associated pathology in vaccinated humans and control viral shedding and transmission.

Evidence is mounting that NAb titers are the immunological correlate of protection for SARS-CoV-2 (Addetia et al., 2020; Yu et al., 2020), and it is increasingly accepted that a successful SARS-CoV-2 vaccine will need to induce potent NAb responses. We observed notable differences in NAb titers by our vaccine and previously described candidates, although comparisons may be biased by differences in vaccination schedules, assay variability, and inconsistencies in data presentation. Here, SARS-CoV-2 S-I53-50NP-vaccinated macaques neutralized authentic virus with a median ID_50_ of ∼4,000 after the final immunization, while NAb titers induced by adenovirus vector vaccines, including ChAdOx1 (van Doremalen et al., 2020) and Janssen (Mercado et al., 2020), DNA vaccines (Yu et al., 2020) and Inovio (Patel et al., 2020), and the inactivated vaccines Sinopharm (Wang et al., 2020) and Sinovacc (Gao et al., 2020), were at least 10-fold lower. However, NAb titers induced by SARS-CoV-2 S-I53-50NPs were similar to the Moderna mRNA vaccine (Corbett et al., 2020) and lower than the Novavax and Clover Biopharmaceuticals protein vaccines (Guebre-Xabier et al., 2020; Liang et al., 2020).

Besides protecting an individual from COVID-19, a key component of an effective SARS-CoV-2 vaccine will be its ability to prevent viral transmission. Hence, sgRNA levels in the upper and lower airways are valuable endpoints in the evaluation of vaccine candidates. Similar to the Janssen and Novavax vaccines, vaccination with SARS-CoV-2 S-I53-50NPs decreased median sgRNA to undetectable levels in the upper airways of all vaccinated animals within 5 dpe, while several other vaccines were unsuccessful in achieving such an effect (van Doremalen et al., 2020; Patel et al., 2020; Yu et al., 2020). One should consider that the Janssen and Novavax studies used 10- to 100-fold lower challenge doses than used here. On the other hand, our regimen consisted of three immunizations, a number that may not be practical in the context of large-scale vaccination campaigns. However, even though three immunizations were used, the high NAb titers at week 6 indicate that two immunizations may be sufficient to confer protection. As in the Novavax study, the animals here were challenged 2 weeks after the final immunization (i.e., at peak NAb titer). It is therefore difficult to draw conclusions on the durability of the protection, but we note that SARS-CoV-2 memory B cells are expected to remain stable over a long period of time (Gaebler et al., 2021). Collectively, the rapid decrease in sgRNA levels, the low level of viral mutations in the S protein, and the absence of an anamnestic response after challenge emphasize SARS-CoV-2 S-I53-50NP vaccination’s profound ability to control infection and replication.

We propose two factors that may have been responsible for the potent humoral responses and protective efficacy by SARS-CoV-2 S-I53-50NPs. First, the high density of antigen on the I53-50NPs may have facilitated efficient activation of relevant NAb B cell lineages, which is in line with previous studies (Antanasijevic et al., 2020; Brouwer et al., 2019) and supported by B cell activation experiments described here. Second, by using an S protein that has been stabilized in the prefusion state, we have likely improved the conformation of key NAb epitopes (such as the RBD) and decreased the exposure of non-NAb epitopes. Indeed, it has recently been shown that introduction of the two appropriately positioned prolines and removal of the polybasic-cleavage site significantly improved the protective ability of an S protein vaccine (Amanat et al., 2020). Nonetheless, these mutations alone might not be sufficient to generate stable trimeric S proteins, and introducing additional stabilizing mutations, such as the previously described HexaPro mutations (Hsieh et al., 2020; Juraszek et al., 2021), may further improve the SARS-CoV-2 S-I53-50NP-induced humoral responses. Recently, nasal immunization has been shown to dramatically improve SARS-CoV-2 vaccine efficacy over intramuscular dosing (Hassan et al., 2020). Using this alternative administration route may allow SARS-CoV-2 S-I53-50NPs to elicit protective NAb titers in the mucosa and could advance its protective efficacy toward fully sterilizing immunity.

### Limitations of study

The data presented here show that three immunizations with SARS-CoV-2 S-I53-50NPs can induce protective immunity to a high-dose SARS-CoV-2 challenge. There are some limitations in our study that we should note. First, we challenged the macaques 2 weeks after the final immunization, generally corresponding to the peak of NAb titers. We therefore cannot ascertain that our vaccine would be as efficacious in the context of a delayed SARS-CoV-2 exposure. Second, our regimen included three immunizations (i.e., one more than vaccines currently in use or under consideration). Although we observed potent NAb responses after two immunizations, we cannot draw firm conclusions on the protective efficacy after two immunizations. Finally, recent months have seen the emergence of novel SARS-CoV-2 strains that may have the potential to evade NAb responses. Whether our vaccine is able to protect against these mutants warrants further investigation.

## STAR★methods

### KEY RESOURCES TABLE

**Table:** 

REAGENT or RESOURCE	SOURCE	IDENTIFIER
**Antibodies**		

COVA1-18	Brouwer et al., 2020	N/A
COVA2-02	Brouwer et al., 2020	N/A
COVA2-15	Brouwer et al., 2020	N/A
COVA2-39	Brouwer et al., 2020	N/A
COVA1-22	Brouwer et al., 2020	N/A
Goat anti-mouse	Jackson Immunoresearch	Cat# 115-005-003; RRID: AB_2338447
Goat anti-rabbit	Jackson Immunoresearch	Cat# 111-035-144; RRID: AB_2307391
Goat anti-human	Jackson Immunoresearch	Cat# 109-005-003; RRID: AB_2337532
goat anti-Human IgG λ	Southern Biotech	Cat# 2070-01; RRID: AB_2795749
goat anti-Human IgG κ	Southern Biotech	Cat# 2060-01; RRID: AB_2795716
polyclonal macaque IgG	Molecular Innovations	Cat# CY-GF-10MG; RRID: AB_10708230
polyclonal human IgG	NIH AIDS reagent program	Cat# 3957
biotinylated mouse anti-monkey IgG	Southern Biotech	Cat# 4700-08; RRID: AB_2796070
Goat-anti-human IgG-PE	Southern Biotech	Cat# 2040-09; RRID: AB_2795648
Goat-anti human IgA-PE	Southern Biotech	Cat# 2050-09; RRID: AB_2795707
Mouse-anti human IgM-PE	Southern Biotech	Cat# 9020-09; RRID: AB_2796577
purified human C1q	Complement Technologies	Cat# A099
mouse anti-human IgG-PE Cy7 (clone G18-145)	BD PharMingen	Cat# 561298; RRID: AB_10611712
mouse anti-human IgM-APC (clone MHM-88)	Biolegend	Cat# 314510; RRID: AB_493011
SARS-CoV Nucleoprotein Rabbit PAb	SinoBiological	Cat# 40143-T62
Goat anti-Rabbit IgG (H+L) Alexa Fluor Plus 488	Invitrogen	Cat# A32731; RRID: AB_2866491
CD27 PE (clone M-T271)	BD biosciences	Cat# 555441; RRID: AB_395834
CD20 PE-CF594 (clone 2H7)	BD biosciences	Cat# 562295; RRID: AB_11153322
IgG PE-Cy7 (clone G18-145)	BD biosciences	Cat# 561298; RRID: AB_10611712
IgM BV605 (clone MHM-88)	Biolegend	Cat# 314524; RRID: AB_2562374
Fixable Viability Dye eF780	eBioscience	Cat# 65-0865-14
CXCR5 PE-Cy7 (clone MU5UBEE)	eBioscience	Cat# 25-9185-41; RRID: AB_2573539
CD3 BUV395 (clone SP34-3)	BD biosciences	Cat# 564117; RRID: AB_2738603
CD4 BUV496 (clone SK3)	BD biosciences	Cat# 612937; RRID: AB_2870220
CD154 BV421 (clone TRAP1)	BD biosciences	Cat# 563886; RRID: AB_2738466
CD69 BV785 (clone FN50)	Biolegend	Cat# 310931; RRID: AB_2561370
CD40 (clone HB14)	Miltenyi Biotec	Cat# 130-094-133; RRID: AB_10839704

**Bacterial and virus strains**		

SARS-CoV-2 virus (hCoV-19/France/ lDF0372/2020 strain)	Lescure et al., 2020	EPI_ISL_410720 (GISAID ID)
SARS-CoV-2 German isolate (BavPat1/2020)	Christian Drosten	N/A

**Biological samples**		

Sera COVID-19 patients	Brouwer et al., 2020	N/A

**Chemicals, peptides, and recombinant proteins**		

Mass spectrometry grade trypsin	Promega	Cat# V5280
Sequencing grade chymotrypsin	Promega	Cat# V1061
Alpha lytic protease	Sigma Aldrich	Cat# A6362
Acetonitrile, 80%, 20% Water with 0.1% Formic Acid, Optima LC/MS	Fisher Scientific	Cat# 15431423
Water with 0.1% Formic Acid (v/v), Optima LC/MS Grade	Fisher Scientific	Cat# LS118-212
Acetonitrile	Fisher Scientific	Cat# 10489553
Trifluoroacetic acid	Fisher Scientific	Cat# 10155347
Dithiothreitol	Sigma-Aldrich	Cat# 43819
Iodacetamide	Sigma-Aldrich	Cat# I1149
PBS	Thermo Fisher	Cat# 10010023
PEI MAX	Polysciences	Cat# 24765-1
HRP-labeled streptavidin	Biolegend	Cat# 405210
3,3′,5,5′-tetranethylbenzidine	Sigma-Aldrich	Cat# T4444
Squalene Emulsion adjuvant	Polymun Scientific	N/A
Polyinosinic-polycytidylic acid	Invivogen	Cat# vac-pic
MPLA liposomes	Polymun Scientific	https://www.polymun.com/liposomes/reference-projects/
1-Ethyl-3-(3-dimethylaminopropyl) carbodiimide	Thermo Fisher Scientific	Cat# A35391
Sulfo-N-Hydroxysulfosuccinimide	Thermo Fisher Scientific	Cat# A39269
FcγRIIa human ectodomain dimer	Bruce Wines & Mark Hogarth	N/A
FcγRIIIa human ectodomain dimer	Bruce Wines & Mark Hogarth	N/A
Poly-L-Lysine Hydrobromide	Sigma-Aldrich	Cat# P1399
Casein buffer	Thermo Scientific	Cat# 37528
Lipofectamine 2000	Life Technologies	Cat# 11668-019
Penicillin	Sigma-Aldrich	Cat# P3032-10MI
Streptomycin	VWR	Cat# 382-EU-100G
Indo-1	Invitrogen	Cat# I1223
CaCl2	Sigma-Aldrich	Cat# C7902
Ionomycin	Invitrogen	Cat# I24222
Staphylococcal enterotoxin B	Merck	Cat# S4881-1MG
Biotin (500 uM)	GeneCopoeia	Cat# BI001
Streptavidin BB515	BD	Cat# 564453
Streptavidin AF647	Biolegend	Cat# 405237
Streptavidin BV421	Biolegend	Cat# 405226
Invitrogen UltraPure 0,5M EDTA, pH 8.0	Thermo Fisher	Cat# 15575020

**Critical commercial assays**		

SSIV Reverse Transcriptase	Thermo Fisher	Cat# 18090050
Q5 Hot Start DNA Polymerase	NEB	Cat# M0494
Ligation Sequencing Kit	Nanopore	Cat# SQK-LSK109
Nano-Glo Luciferase Assay System	Promega	Cat# N1130
Nucleospin 96 Virus Core	Macherey-Nagel	Cat# 740452.4
Superscript III platinum on step qRT-PCR	Thermo Fischer	Cat# 11732088
Monkey IFN-g ELISPOT pro	Mabtech	Cat# 3421M-2APT
Biotin protein ligase	GeneCopoeia	Cat# BI001

**Experimental models: cell lines**		

FreeStyle 293F cells	Thermo Fisher	Cat# R79007
HEK293T/ACE2 cells	Schmidt et al., 2020	N/A
HEK293T cells	ATCC	Cat# CRL-11268
Ramos B cells	Obtained through the NIH AIDS Reagent Program, Division of AIDS, NIAID, NIH; from Drs. L. Wu and V. N. KewalRaman	N/A
VeroE6	ATCC	ATCC® CRL 1586TM

**Experimental models: organisms/strains**		

BALB/cAnNCrl mice	Charles River Laboratories	N/A
New Zealand White rabbits	Covance Research Products, Inc	N/A
Cynomolgus macaques	Noveprim	N/A

**Oligonucleotides**		

Primers covid19 V3	ARTIC network	https://artic.network/resources/ncov/ncov-amplicon-v3.pdf
RdRp-IP4 primers F- GGT AAC TGG TAT GAT TTC G, R - CTG GTC AAG GTT AAT ATA GG, probe P - TCA TAC AAA CCA CGC CAG G	https://www.who.int/docs/default-source/coronaviruse/real-time-rt-pcr-assays-for-the-detection-of-sars-cov-2-institut-pasteur-paris.pdf?sfvrsn=3662fcb6_2	N/A
sgLeadSARSCoV2-F CGATCTCTTGTAGATCTGTTCTC, E-Sarbeco-R primer ATATTGCAGCAGTACGCACACA, E-Sarbeco probe HEX-ACACTAGCCATCCTTACTGCGCTTCG-BHQ1	Corman et al., 2020	N/A

**Recombinant DNA**		

pHIV-1_NL43_ΔENV-NanoLuc plasmid	Schmidt et al., 2020	N/A
SARS-CoV-2-S_Δ19_ plasmid	Schmidt et al., 2020	N/A
SARS-CoV-2 S pPPI4 plasmid	Brouwer et al., 2020	N/A
SARS-CoV-2 S-I53-50A.1NT1 pPPI4 plasmid	This study	N/A
SARS-CoV-2 RBD pPPI4 plasmid	Brouwer et al., 2020	N/A
SARS-CoV-2 S-AVI pPPI4 plasmid	This Study	N/A
SARS-CoV-2 RBD-AVI pPPI4 plasmid	This Study	N/A
pRRL EuB29 gl2-1261 IgGTM.BCR.GFP.WPRE plasmid	McGuire et al., 2014	N/A
gblock COVA1-18 & COVA2-15	Integrated DNA Technologies	N/A
pMDLg	Dull et al., 1998	Addgene Cat# 12251
pRSV-Rev	Dull et al., 1998	Addgene Cat# 12259
pVSV-g	Gee et al., 2020	Addgene Cat# 138479

**Software and algorithms**		

Empower 3.0	Waters	N/A
Masslynx v4.1	Waters	N/A
Driftscope version 2.8	Waters	N/A
Byos™ (Version 3.9)	Protein Metrics Inc.	N/A
GraphPad Prism v8	GraphPad	N/A
XCalibur Version v4.2	Thermo Fisher	N/A
Orbitrap Fusion Tune application v3.1	Thermo Fisher	N/A
Flowjo v10	Flowjo	N/A
UCSF ChimeraX	Goddard et al., 2018	N/A
GraphPad Prism v7	GraphPad	N/A
Canu	Koren et al., 2017	https://github.com/marbl/canu
Minimap2	Li, 2018	https://github.com/lh3/minimap2
Varscan	Koboldt et al., 2012	http://varscan.sourceforge.net/
Longshot	Edge and Bansal, 2019	https://github.com/pjedge/longshot
INTELLISPACE PORTAL 8 software	Philips healthcare	https://www.philips.sa/en/healthcare/product/HC881062/intellispace-portal-80-all-your-advanced-analysis-needs-one-comprehensive-solution
ImageQuant TL 8.2 image analysis software	GE Healthcare	N/A

**Other**		

EasySpray PepMap RSLC C18 column (75 μm x 75 cm)	Thermo Fisher Scientific	Cat# ES805
C18 ZipTip	Merck Milipore	Cat# ZTC18S008
Vivaspin 500, 3 kDa MWCO, Polyethersulfone	Sigma-Aldrich	Cat# GE28-9322-18
PepMap100 C18 3UM 75UMx2CM Nanoviper	Thermo Scientific	Cat# 164946
Ni-NTA agarose	QIAGEN	Cat# 30210
Ni-NTA HighSorb plates	QIAGEN	Cat# 35061
NuPAGE 4-12% Bis-Tris gels	Thermo Fisher	Cat# NP0321BOX
Superose 6 increase 10/300 GL	Sigma-Aldrich	Cat# GE29-0915-96
Econo-column chromatography columns	BIO RAD	Cat# 7371512
NGC chromatography system	BIO RAD	N/A
Octet K2 system	Sartorius (FortéBio)	N/A
Octet Biosensors: Protein A	Sartorius (FortéBio)	Cat# 18-5010
Vivaspin 20, 100.000 kDa MWCO, Polyethersulfone	Sigma-Aldrich	Cat# GE28-9323-63
Nucleobond Xtra Maxi kit	Macherey-Nagel	Cat# 740414.50
Fast Digest BamHI	Thermo Scientific	Cat# FD0054
Fast Digest Green buffer 10x	Thermo Scientific	Cat# B72
Fast Digest PstI	Thermo Scientific	Cat# FD0614
Magplex Microspheres	Luminex	Cat# MC10043-01
Streptavidin-PE	Thermo Fisher Scientific	Cat# 12-4317-87
Bioplex 200	Bio-Rad	Cat# 171000205
FreeStyle 293 Expression medium	Thermo Scientific	Cat# 12338018
DMEM	Sigma-Aldrich	Cat# D6429-500ML
Glutamax supplement	Thermo Fisher	Cat# 35050061
High-binding plates: Half-area 96-well polystyrene high-binding microplate	Greiner	Cat# 675061
Steritop Filter Units	Merckmillipore	Cat# C3239
Glomax	Turner BioSystems	Model# 9101-002
Microplate 96 well half area white	Greiner bio-one	Cat# 675074
VTM medium	CDC, DSR-052-01	https://www.cdc.gov/coronavirus/2019-ncov/downloads/Viral-Transport-Medium.pdf
RPMI Medium 1640	GIBCO	Cat# 21875-034
Hanks’ Balanced Salt Solution (HBSS)	GIBCO	Cat# 14175-053
FACS Aria-II SORP	BD biosciences	N/A
LSR Fortessa	BD biosciences	N/A
Amersham Typhoon Biomolecular Imager	GE Healthcare	Amersham Typhoon Biomolecular Imager
BD micro-fine+; U-100 Insulin 0.5 ml; 0,33 (29G)x12,7 mm	BD biosciences	Cat# 037-7614
Microvette CB300 K2E	Sarstedt	Cat# 16.444.100
HEPES (1M, GIBCO)	Thermofisher scientific	Cat# 15630106
Greiner CELLSTAR® 96 well plates round bottom clear wells	Merck	Cat# M9436
Lemo21 (DE3)	New England Biolabs	Cat# C2528J
Ni Sepharose 6 FF	Cytiva	Cat#17531808
AKTA Avant150 FPLC system	Cytiva	N/A
Superdex 200 Increase SEC column	Cytiva	Cat# 28-9909-44

### Resource availability

#### Lead contact

Further information and requests for resources and reagents should be directed to and will be fulfilled by the lead contact, Rogier W. Sanders (r.w.sanders@amsterdamumc.nl).

#### Materials availability

All reagents will be made available on request after completion of a Materials Transfer Agreement.

#### Data and code availability

The raw data supporting the findings of the study are available from the corresponding authors upon reasonable request.

### Experimental model and subject details

#### Cell lines

HEK293F (Life Technologies) and HEK293T (ATCC CRL-11268) are human embryonic kidney cell lines transformed for increased production of recombinant protein or retrovirus. HEK293F cells are adapted to grow in suspension. HEK293F cells were cultured at 37°C with 8% CO_2_ and shaking at 125 rpm in 293FreeStyle expression medium (Life Technologies). HEK293T cells were cultured at 37°C with 5% CO_2_ in flasks with DMEM supplemented with 10% fetal bovine serum (FBS), streptomycin (100 μg/mL) and penicillin (100 U/mL). VeroE6 (ATCC CRL-1586) is a kidney epithelial cell from African green monkeys. VeroE6 cells were cultured at 37°C with 5% CO_2_ in DMEM supplemented with or without streptomycin (100 μg/mL) and penicillin (100 U/mL) and with or without 5 or 10% FBS, and with or without TPCK-trypsin. HEK293T/ACE2 cells (Schmidt et al., 2020) are a human embryonic kidney cell line expressing human angiotensin-converting enzyme 2. HEK293T/ACE2 cells were cultured at 37°C with 5% CO_2_ in flasks with DMEM supplemented with 10% FBS, streptomycin (100 μg/mL) and penicillin (100 U/mL). Ramos is a human Burkitt’s lymphoma cell line that has B lymphocyte characteristics. Ramos B cells were cultured at 37°C with 5% CO_2_ in RPMI supplemented with 10% FBS, streptomycin (100 μg/mL) and penicillin (100 U/mL).

#### Cynomolgus macaques

Female cynomolgus macaques (*Macaca fascicularis*), aged 56-66 months and originating from Mauritian AAALAC certified breeding centers were used in this study. All animals were housed in IDMIT infrastructure facilities (CEA, Fontenay-aux-roses), under BSL-2 and BSL-3 containment when necessary (Animal facility authorization #D92-032-02, Préfecture des Hauts de Seine, France) and in compliance with European Directive 2010/63/EU, the French regulations and the Standards for Human Care and Use of Laboratory Animals, of the Office for Laboratory Animal Welfare (OLAW, assurance number #A5826-01, US). The protocols were approved by the institutional ethical committee “Comité d’Ethique en Expérimentation Animale du Commissariat à l’Energie Atomique et aux Energies Alternatives” (CEtEA #44) under statement number A20-011. The study was authorized by the “Research, Innovation and Education Ministry” under registration number APAFIS#24434-2020030216532863v1.

#### Rabbits

Female New Zealand White rabbits of 2.5-3 kg from multiple litters were used. Animals were sourced and housed at Covance Research Products, Inc. (Denver, PA, USA) and immunizations were performed under permits with approval number C0084-20. Immunization procedures complied with all relevant ethical regulations and protocols of the Covance Institutional Animal Care and Use Committee.

#### Mice

Female BALB/cAnNCrl mice, aged 8 weeks, were ordered from Charles River Laboratories and housed at the Animal Research Institute Amsterdam under BSL-2 conditions. All experiments were performed in accordance with the Dutch Experiment on Animals Act and were approved by the Animal Ethics Committee of the Amsterdam UMC (Permit number 17-4045).

#### Patient sera

Sera were collected from male and female patients aged between 18 and 75 years through the COVID-19 Specific Antibodies (COSCA) study, which was performed at the Amsterdam University Medical Centre, location AMC, the Netherlands under approval of the local ethical committee of the AMC (NL 73281.018.20) (Brouwer et al., 2020).

### Method details

#### Construct design

To create the SARS-CoV-2 S-I53-50A.1NT1 construct, the previously described pPPI4 plasmid encoding the prefusion stabilized SARS-CoV-2 S protein (Brouwer et al., 2020) was digested with PstI and BamHI and ligated in a PstI-BamHI-digested pPPI4 plasmid encoding a modified I53-50A.1NT1 sequence. The original I53-50A.1NT1 plasmid was described previously (Brouwer et al., 2019). Modifications constitute the introduction of GSLEHHHHHH after the final residue to introduce a C-terminal histidine-tag. To generate S proteins for FACS analyses, the prefusion S protein sequence was cloned into a pPPI4 backbone encoding a I53-50A.1NT1 sequence that had an Avi- and histidine-tag after the final residue. The RBD probe was generated by digesting this plasmid with PstI-BamHI and introducing, by Gibson assembly, a gene encoding the RBD (residues 319-541) directly downstream of a TPA leader sequence. For ELISAs, Luminex assays, and ELISpots, histidine-tagged versions of SARS-CoV-2 S protein and RBD were produced by cloning the aforementioned sequences into a pPPI4 plasmid containing a hexahistidine tag using PstI and BamHI digestion and ligation.

#### Protein expression and purification

All constructs were expressed by transient transfection of HEK293F cells (Invitrogen) maintained in Freestyle medium (Life Technologies) at a density of 0.8-1.2 million cells/mL. On the day of transfection, a mix of PEImax (1 μg/μL) with expression plasmids (312.5 μg/L) in a 3:1 ratio in OptiMEM (GIBCO) were added to the cells. Six days post transfection, supernatants were centrifuged for 30 min at 4000 rpm, filtered using 0.22 μm Steritop filters (Merck Millipore), and subjected to affinity purification using Ni-NTA agarose beads. Protein eluates were then concentrated and buffer exchanged to PBS using Vivaspin filters with the appropriate molecular weight cutoff (GE Healthcare). Protein concentrations were determined by the Nanodrop method using the proteins peptidic molecular weight and extinction coefficient as determined by the online ExPASy software (ProtParam).

#### I53-50B.4PT1 expression and purification

Lemo21 (DE3) (NEB) cells expressing I53-50B.4PT1 were grown in a 10L BioFlo 320 Fermenter (Eppendorf) or a 2L shake flask. Cells were grown in LB (10 g Tryptone, 5 g Yeast Extract, 10 g NaCl) at 37°C to an OD600 of 0.8. After the cells were induced with 1 mM of IPTG, temperature was reduced to 18°C and cells were grown for 16h. The cells were then lysed in 50 mM Tris, 500 mM NaCl, 30 mM imidazole, 1 mM PMSF, 0.75% CHAPS using a Microfluidics M110P at 18,000 psi. Lysate was centrifuged at 24,000 g for 30 minutes. Clarified lysate was next applied to a Ni Sepharose 6 FF column (Cytiva) linked to an AKTA Avant150 FPLC system (Cytiva) for immobilized metal affinity chromatography. I53-50B.4PT1 was eluted using a 30 mM to 500mM imidazole linear gradient in 50 mM Tris pH 8, 500 mM NaCl and 0.75% CHAPS. Fractions containing I53-50B.4PT1 were pooled, concentrated using centrifugal filters with a 10,000 kDa cutoff (Millipore), sterilized and applied to a Superdex 200 Increase 10/300 (Cytvia) for further purification. Batches were tested for endotoxin levels before use.

#### SARS-CoV-2 S-I53-50NP assembly

Ni-NTA eluates of SARS-CoV-2 S-I53-50A.1NT1 (expressed as described above) were buffer exchanged to Tris-buffer-saline (TBS), sterile filtered (0.22 μm) and applied to a Superose 6 increase 10/300 GL column (GE healthcare) linked to a NGC chromatography system (Bio-Rad) in TBS supplemented with 5% glycerol. Size exclusion fractions between 12-14 mL were pooled and an equimolar amount of I53-50B.4PT1 was added. After an overnight incubation at 4°C, the assembly mix was applied to a Superose 6 increase 10/300 GL column in TBS+5% glycerol to remove unassembled components. Fractions corresponding to assembled SARS-CoV-2 S-I53-50NPs (8.5-9.5 mL) were collected and concentrated using Vivaspin columns with a molecular weight cutoff of 10,000 kDa (GE Healthcare). Protein concentrations were determined by the Nanodrop method using the proteins peptidic molecular weight and extinction coefficient as determined by the online ExPASy software (ProtParam).

#### BN-PAGE analysis

BN-PAGE was performed as described previously (de Taeye et al., 2015). Briefly, 2.5 μg of S protein was mixed with loading dye and run on a 4%–12% Bis-Tris NuPAGE gel (Invitrogen).

#### Negative-stain EM

SARS-CoV-2 S-153-50NPs were added to carbon-covered 400 mesh copper grids and stained with 2% uranyl formate. Micrographs were imaged on a Tecnai F12 Spirit microscope with a 4k FEI Eagle CCD. Leginon (Potter et al., 1999) and Appion (Lander et al., 2009) were used to collect and process micrographs.

#### BLI assay

SARS-CoV-2 S-I53-50A.1NT1 and SARS-CoV-2 S-I53-50NP were diluted to 100 nM and 5nM, respectively, in BLI running buffer (PBS/0.1% bovine serum albumin/0.02% Tween20) and antibody binding was assessed using a ForteBio Octet K2. The binding assays were performed at 30°C and with agitation set at 1000 rpm. Antibody was loaded on protein A sensors (ForteBio) at 10 μg/mL in running buffer until a binding threshold of 1 nm was reached. Association and dissociation were measured for 300 s.

#### Glycopeptide analysis by liquid chromatography-mass spectrometry

Gycopeptide analysis was essentially performed as described previously (Watanabe et al., 2020). A 30 μg aliquot of SARS-CoV-2 S-I53-50A.1NT1 was denatured, alkylated, reduced and cleaved by the proteases trypsin, chymotrypsin and alpha-lytic protease. Clean-up of the resulting peptide/glycopeptides was performed using C18 Zip-tips and the sample was analyzed by nanoLC-ESI MS with an Easy-nLC 1200 (Thermo Fisher Scientific) system coupled to a Fusion mass spectrometer (Thermo Fisher Scientific) using higher energy collision-induced dissociation (HCD) fragmentation. Peptides were separated using an EasySpray PepMap RSLC C18 column (75 μm × 75 cm). Glycopeptide fragmentation data were extracted from the raw file using Byonic™ (Version 3.5) and Byologic™ software (Version 3.5; Protein Metrics Inc.).

#### Generation of B cells that stably express COVID-specific B cell receptors

The B cell specific expression plasmid was constructed by exchanging the gl2-1261 gene of the pRRL EuB29 gl2-1261 IgGTM.BCR.GFP.WPRE plasmid (McGuire et al., 2014) with heavy and light chain genes of either COVA1-18 and COVA2-15 using Gibson assembly (Integrated DNA Technologies). The production of lentivirus in HEK293T and the subsequent transduction was conducted as described elsewhere (ter Brake et al., 2006). In short, lentiviruses were produced by co-transfecting the expression plasmid with pMDL, pVSV-g and pRSV-Rev into HEK293T cells using lipofectamine 2000 (Invitrogen). Two days post transfection, IgM-negative Ramos B cells cultured in RPMI (GIBCO) supplemented with 10% fetal calf serum, streptomycin (100 μg/mL) and penicillin (100 U/mL) (RPMI++) were transduced with filtered (0.45 μm) and concentrated (100 kDa molecular weight cutoff, GE Healthcare) HEK293T supernatant. Seven days post transduction, BCR-expressing B cells were FACS sorted on IgG and GFP double-positivity using a FACS Aria-II SORP (BD biosciences). B cells were then expanded and cultured indefinitely.

#### B cell activation assay

B cell activation experiments of SARS-CoV-2 S protein-specific Ramos B cells were performed as previously described (Brouwer et al., 2019; Sliepen et al., 2019). In short, 4 million cells/mL in RPMI++ were loaded with 1.5 μM of the calcium indicator Indo-1 (Invitrogen) for 30 min at 37°C, washed with Hank’s Balance Salt Solution supplemented with 2 mM CaCl_2_, followed by another incubation of 30 min at 37°C. Antigen-induced Ca^2+^ influx of COVID-specific B cells were monitored on a LSR Fortessa (BD Biosciences) by measuring the 379/450 nm emission ratio of Indo-1 fluorescence upon UV excitation. Following 30 s of baseline measurement, aliquots of 1 million cells/mL were then stimulated for 100 s at RT with either 5 μg/mL, 1 μg/mL, 200 ng/mL or 40 ng/mL of SARS-CoV-2 S-I53-50A.1NT1 or the equimolar amount presented on I53-50NPs. Ionomycin (Invitrogen) was added to a final concentration of 1 mg/mL to determine the maximum Indo-1-fluorescence. Kinetics analyses were performed using FlowJo v10.7.

#### Animals and study designs

Cynomolgus macaques were randomly assigned in two experimental groups. The vaccinated group (n = 6) received 50 ug of SARS-CoV-2 S-I53-50NP adjuvanted with 500 μg of MPLA liposomes (Polymun Scientific, Klosterneuburg, Austria) diluted in PBS at weeks 0, 4 and 10, while control animals (n = 4) received no vaccination. Vaccinated animals were sampled in blood, nasal swabs and saliva at weeks 0, 2, 4, 6, 8, 10 and 12. At week 12, all animals were exposed to a total dose of 10^6^ pfu of SARS-CoV-2 virus (hCoV-19/France/ lDF0372/2020 strain; GISAID EpiCoV platform under accession number EPI_ISL_406596) via the combination of intranasal and intra-tracheal routes (0,25 mL in each nostril and 4,5 mL in the trachea, i.e., a total of 5 mL; day 0), using atropine (0.04 mg/kg) for pre-medication and ketamine (5 mg/kg) with medetomidine (0.042 mg/kg) for anesthesia. Saliva, as well as nasopharyngeal, tracheal and rectal swabs, were collected at days 1, 2, 3, 4, 5, 6, 10, 14 and 21 days past exposure (dpe) while blood was taken at days 2, 4, 6, 10, 14 and 21 dpe. Bronchoalveolar lavages (BAL) were performed using 50 mL sterile saline on 3 dpe. A single BAL was performed at 3 dpe in order to be close to the peak of viral replication and to be able to observe a difference between the vaccinated and control groups. In our earlier study (Maisonnasse et al., 2020), we found that at later time-points, viral loads in the BAL were very low or negative. Chest CT was performed at baseline and on 3, 7 and 14 dpe in anesthetized animals using tiletamine (4 mg/kg) and zolazepam (4 mg/kg). Blood cell counts, haemoglobin and hematocrit were determined from EDTA blood using a HMX A/L analyzer (Beckman Coulter).

Female New Zealand White rabbits were given two intramuscular immunizations, one in each quadricep, at weeks 0, 4, and 12. The immunization mixture involved 39 μg of SARS-CoV-2-S-I53-50NPs (equal to 30 μg of SARS-CoV-2-S) formulated 1:1 in Squalene Emulsion adjuvant (Polymun, Klosterneuburg, Austria). The rabbits were bled on the day of immunization and then at weeks 2, 4, 6, and 14.

Female Balb/cAnNCrl mice received subcutaneous immunizations into the neck skin-fold at weeks 0, 4, and 12. The immunization mixture contained 13 μg of SARS-CoV-2-S-I53-50NPs (equal to 10 μg of SARS-CoV-2-S) adjuvanted with 50 μg of polyinosinic-polycytidylic acid (Poly-IC; Invivogen). Blood was collected at weeks −1, 2, 6 and 14. Two out of eight mice were sacrificed at week 6 to allow interim analysis of the induced humoral response.

#### Patient samples

Patients with at least one nasopharyngeal swab positive for SARS-CoV-2 as determined by qRT-PCR (Roche LightCycler480, targeting the Envelope-gene 113bp) were included. Patients using immunosuppressive mediation (equivalent of > 7.5 mg prednisolone) were excluded from the study. Included patients signed written informed consent forms. A venipuncture was performed to obtain blood in Acid Citrate Dextrose tubes for serum collection approximately four weeks after onset of COVID-19 symptoms (Brouwer et al., 2020).

#### ELISAs

Ni-NTA plates (QIAGEN) were loaded with 2 μg/mL of SARS-CoV-2 S protein in TBS for 2 h at RT. Next, washed plates were blocked for 1 h with TBS/2% skimmed milk. Four-fold serial dilutions of inactivated sera, starting from a 1:200 dilution for mice and rabbit sera, and 1:100 dilution for macaque sera, were added in TBS/2% skimmed milk/20% sheep serum and incubated for 2 h at RT. Following plate washing, a 1:3000 dilution of secondary antibody in TBS/2% skimmed milk was added for 1 h at RT. For mice, rabbit and macaque sera horseradish peroxidase (HRP)-labeled goat anti-mice IgG, HRP-labeled goat anti-rabbit IgG and HRP-labeled goat anti-human IgG (Jackson Immunoresearch) was used, respectively. Finally, after washing plates with TBS/0.05% Tween-20, developing solution (1% 3,3′,5,5′-tetranethylbenzidine (Sigma-Aldrich), 0.01% H_2_O_2_, 100 mM sodium acetate and 100 mM citric acid) was added to each well and a colorimetric endpoint was obtained by adding 0.8 M H_2_SO_4_ after 1.5 min. Binding endpoint titers were determined using a cutoff of 5x background.

As affinity differences between species-specific secondary Abs may bias comparisons between endpoint titers of macaque and convalescent human sera, we used a different ELISA setup to compare S protein and RBD binding responses. Specifically, ELISA binding responses are compared to a standard curve of species-specific polyclonal IgG so that a semiquantitative measure of specific IgG concentrations can be obtained. High-binding plates were direct-coated overnight at 4°C with 2 μg/mL of SARS-CoV-2 S or 0.4 μg/mL of SARS-CoV-2 RBD in PBS. For capture of the standard curve (i.e., macaque and human polyclonal IgG), plates were coated with 1:2000 (for capture of polyclonal macaque IgG) or 1:1000 (for capture of polyclonal human IgG) of goat anti-Human IgG λ and κ (Southern Biotech). The next day, plates were washed with TBS than contained 0.05% Tween-20 (TBST) and blocked for 1 h with Casein buffer (Thermo Fisher Scientific). A 1:100, 1:1000 and 1:10,000 dilution of macaque or human serum in Casein buffer were then added to the wells containing S protein or RBD. Five-fold serial dilutions of polyclonal macaque (Molecular Innovations) or human IgG, starting at a concentration of 1 μg/mL, were added to wells containing the coated goat anti-Human IgG λ and κ. Standards and samples were applied in duplicate. Following a 1 h incubation at RT, the plates were washed with TBST and secondary antibody diluted in Casein buffer was added for 1 h. For the plates containing human sera and standard, a 1:3000 dilution of HRP-labeled goat anti-human IgG (Jackson Immunoresearch) was used. For plates containing macaque sera and standard, a 1:50,000 dilution of biotinylated mouse anti-monkey IgG was used (Southern Biotech), followed by a 1 h incubation with a 1:3000 dilution of HRP-labeled streptavidin (Biolegend) in Casein. Finally, after washing plates with TBST, developing solution (1% 3,3′,5,5′-tetranethylbenzidine (Sigma-Aldrich), 0.01% H_2_O_2_, 100 mM sodium acetate and 100 mM citric acid) was added to each well and a colorimetric endpoint was obtained by adding 0.8 M H_2_SO_4_ after 5 min. OD_450_-values that fell within the linear range of the standard curve were fitted and the concentration of SARS-CoV-2 S protein and RBD-specific IgG was determined.

#### Pseudovirus neutralization assay

To generate SARS-CoV-2 pseudovirus, HEK293T cells (ATCC, CRL-11268) were transfected with a pHIV-1_NL43_ΔENV-NanoLuc reporter virus plasmid and a SARS-CoV-2-S_Δ19_ plasmid (Schmidt et al., 2020). Cell supernatant containing the pseudoviruses was harvested 48 h post transfection, centrifuged for 5 min at 500 x *g* and sterile filtered through a 0.22 μm pore size PVDF syringe filter. For neutralization assays HEK293T expressing the SARS-CoV-2 receptor ACE2 (HEK293T/ACE2) were cultured in DMEM (GIBCO), supplemented with 10% FBS, penicillin (100 U/mL), and streptomycin (100 μg/mL).To determine the neutralization activity in serum, saliva or nasopharyngeal samples, HEK293T/ACE2 cells were first seeded in 96-well plates coated with 50 μg/mL poly-l-lysine at a density of 2x10^4^/well in the culture medium described above but with GlutaMax (GIBCO) added. The next day, duplicate serial dilutions of heat inactivated samples were prepared in the same medium as used for seeding of cells and mixed 1:1 with pseudovirus. This mixture was incubated at 37°C for 1 h before adding it to the HEK293T/ACE2 cells in a 1:1 ratio with the cell culture medium. After 48 h, the cells were lysed and lysate was transferred into half area 96-well white microplates (Greiner bio-one). Luciferase activity was measured in the lysates using the Nano-Glo Luciferase Assay System (Promega) with a Glomax system (Turner BioSystems). Relative luminescence units (RLU) were normalized to those from cells infected with SARS-CoV-2 pseudovirus in the absence of sera/saliva/swabs. Neutralization titers (ID_50_-values) were determined as the serum dilution at which infectivity was inhibited by 50%.

#### Authentic virus neutralization assay

Serum samples were tested for their neutralization activity against SARS-CoV-2 (German isolate; GISAID ID EPI_ISL 406862; European Virus Archive Global #026V-03883) as described previously (Okba et al., 2020). In short, serum samples were serially diluted in 50 μL Opti-MEM I (GIBCO), supplemented with GlutaMAX (GIBCO) and penicillin (100 U/mL). Serum dilutions were mixed with 400 plaque-forming units of virus to a total of 100 μL. This mixture was incubated at 37°C for 1 h. After incubation, the mixtures were put on Vero-E6 cells (ATCC CRL-1586) and incubated for 1 h. Cells were then washed, Opti-MEM I supplemented with GlutaMAX (GIBCO) was added, and incubated for 8 h. Cells were fixed with 4% formaldehyde in PBS and stained with polyclonal rabbit anti-SARS-CoV Ab (Sino Biological) and secondary Alexa488 conjugated goat-anti-rabbit Ab (Invitrogen). Plates were scanned on the Amersham Typhoon Biomolecular Imager (channel Cy2; resolution 10 μm; GE Healthcare). Data was analyzed using ImageQuant TL 8.2 image analysis software (GE Healthcare).

#### Protein coupling to Luminex beads

Proteins were covalently coupled to Magplex beads (Luminex Corporation) using a two-step carbodiimide reaction and a ratio of 75 μg protein SARS-CoV-2 S protein to 12,5 million beads. Magplex beads (Luminex Corporation) were washed with 100 mM monobasic sodium phosphate pH 6.2, activated for 30 min on a rotor at RT by addition of Sulfo-N-Hydroxysulfosuccinimide (Thermo Fisher Scientific) and 1-Ethyl-3-(3-dimethylaminopropyl) carbodiimide (Thermo Fisher Scientific). The activated beads were washed three times with 50 mM MES pH 5.0 and added to SARS-CoV-2 S protein which was diluted in 50 mM MES pH 5.0. The beads and protein were incubated for 3 h on a rotator at RT. Afterward, the beads were washed with PBS and blocked with PBS containing 0.1% BSA, 0.02% Tween-20 and 0.05% Sodium Azide at pH 7.0 for 30 min on a rotator at RT. Finally, the beads were washed and stored in PBS containing 0.05% Sodium Azide at 4°C and used within 3 months.

#### Luminex assays

50 μL of a working bead mixture containing 20 beads per μL was incubated overnight at 4°C with 50 μL of diluted serum, saliva or nasopharyngeal swab. Pilot experiments determined the optimal dilution for detection of SARS-CoV-2 S protein-specific IgG, IgA and IgM antibodies in serum to be 1:50,000, for the detection of antibody binding to FcγRIIa, FcγRIIIa and C1q in serum at 1:500 and for detection of S protein-specific IgG and IgA in saliva and nose fluid at 1:20. Plates were sealed and incubated on a plate shaker overnight at 4°C. The next day, plates were washed with TBS containing 0.05% Tween-20 (TBST) using a hand-held magnetic separator. Beads were resuspended in 50 μL of Goat-anti-human IgG-PE, Goat-anti human IgA-PE or Mouse-anti human IgM-PE (Southern Biotech) and incubated on a plate shaker at RT for 2 h. For C1q binding, beads were resuspended in 50 μL purified human C1q (Complement Technology, Inc.), which was biotinylated and conjugated to Streptavidin-PE. For the detection of FcγRIIa and FcγRIIIa binding, beads were resuspended in 50 μL biotinylated human FcγRIIa and FcγRIIIa ectodomain dimers (courtesy of Bruce Wines and Mark Hogarth) for 2 h incubation, after which the beads were washed with TBST and then incubated with 50 μL Streptavidin-PE (Invitrogen) on a plate shaker for 1 h. Afterward, the beads were washed with TBST and resuspended in 110 μL Bioplex sheath fluid (Bio-Rad). The beads were agitated for a few minutes on a plate shaker at RT and then readout was performed on the Bioplex 200 (Bio-Rad). Resulting mean fluorescence intensity (MFI) values were corrected by subtraction of MFI values from buffer and beads only wells. Relative MFI values were obtained by dividing the MFI by the background MFI at week 0. Reproducibility of the results was confirmed by performing replicate runs.

#### SARS-CoV-2 S protein-specific CD4- and cTfh cell analysis using an activation induced marker (AIM) assay

Cryopreserved macaque PBMCs from 2 weeks after final immunization (week 12) were thawed and counted. 1x10^6^ cells per condition were plated in 96 U-shape well plates (CELLSTAR, Kremsmünster) and rested for 2-3 h in RPMI (GIBCO), supplemented with 10% FBS, 1mM HEPES (GIBCO), penicillin (100 U/mL), and streptomycin (100 μg/mL). Subsequently, cells were incubated with 0.5 mg/mL anti-CD40 (Miltenyi Biotec) for 15 min at 37°C, to prevent downregulation of CD40 ligand (CD40L) by blocking CD40-CD40L interaction. PBMCs were stimulated for 18 h at 37°C with 5 mg/mL SARS-CoV-2 S protein. As a negative control no stimulants were added, and as a positive control 1 mg/mL Staphylococcal enterotoxin B was used. Thereafter, PBMCs were washed using FACS buffer (PBS, supplemented with 1% FBS). PBMCs were then incubated with the cTfh-specific chemokine receptor antibody CXCR5 PE-Cy7 (clone MU5UBEE; eBioscience) for 10 min at 37°C. Next, the following antibodies and viability dye were incubated with PBMCs at RT for 20 min: anti-CD3 BUV395 (clone SP34-2; BD Biosciences), anti-CD4 BUV495 (clone SK3; BD Biosciences), anti-CD154 BV421 (clone TRAP1; BD Biosciences), anti-CD69 BV785 (clone FN50; BD Biosciences), anti-4-1BB APC (clone 4B4-1; Biolegend) and LiveDead-eF780 (eBioscience). Cells were washed twice with FACS buffer before acquisition on the BD LSR II Fortessa 5 lasers flow cytometer (BD biosciences). Analysis was performed on FlowJo v.10.7.1.

#### SARS-CoV-2 S protein and RBD-specific B cell analysis

Biotinylated SARS-CoV-2 S protein and RBD were conjugated to streptavidin-bound fluorophores. Briefly, the biotinylated proteins were incubated for a minimum of 1 h at 4°C with the streptavidin-conjugates AF647 (Biolegend), BV421 (Biolegend) and BB515 (BD Biosciences) at a 1:2 protein to fluorochrome ratio. The fluorescent probes were incubated for at least 10 min with 10mM biotin (GeneCopoeia) to saturate the unconjugated streptavidin-fluorochrome complexes. Cryopreserved macaque PBMCs from 2 weeks after final immunization (week 12) were thawed and counted. 5x10^6^ cells were stained for 30 min at 4°C with the fluorescent probes, a viability marker (LiveDead-eF780, eBiosciences) and the following B cell-specific antibodies: anti-CD20-PE-CF594 (clone 2H7; BD Biosciences), anti-IgG-PE-Cy7 (clone G18-145;BD Biosciences), anti-CD27-PE (clone M-T271; BD Biosciences), and anti-IgM-BV605 (clone MHM-88; Biolegend). Cells were washed twice with FACS buffer and acquired on the FACS-ARIA-SORP 4 laser (BD Biosciences). Analysis was performed on FlowJo v.10.7.1.

#### Viruses and cells

For the cynomolgus macaque challenge study, SARS-CoV-2 virus (hCoV-19/France/ lDF0372/2020 strain; GISAID EpiCoV platform under accession number EPI_ISL_406596) was isolated by the National Reference Center for Respiratory Viruses (Institut Pasteur, Paris, France) (Lescure et al., 2020). Briefly, Vero E6 cells were cultured in DMEM + 5% FBS. PCR-positive sample from patient 0371 was diluted 1:2 with DMEM with antibiotics but without FBS and supplemented with 1ug/ml trypsin-TPCK (Sigma-Aldrich), added to the cells and incubated one hour at 37°C. The inoculum was then replaced with DMEM medium containing antibiotics and 1μg/ml trypsin-TPCK at 37°C. Three days later, clear cytopathogenic effects were observed and RT-qPCR was performed to detect presence of virus. Culture supernatant was harvested, aliquoted and stored at −80°C.

Before the challenge experiment, virus stocks were first subjected to an additional passage on Vero E6 cells in DMEM (GIBCO) without FBS, supplemented with penicillin (100 U/mL), streptomycin (100 μg/mL), and 1 μg/mL TPCK-trypsin at 37°C in a humidified CO2 incubator. Virus was then titrated on Vero E6 cells by plaque reduction assay.

#### Virus quantification in cynomolgus macaque samples

Upper respiratory (nasopharyngeal and tracheal) and rectal specimens were collected with swabs (Viral Transport Medium, CDC, DSR-052-01). Tracheal swabs were performed by insertion of the swab above the tip of the epiglottis into the upper trachea at approximately 1.5 cm of the epiglottis. All specimens were stored between 2°C and 8°C until analysis by RT-qPCR with a plasmid standard concentration range containing an RdRp gene fragment including the RdRp-IP4 RT-PCR target sequence. SARS-CoV-2 E gene subgenomic mRNA (sgmRNA) levels were assessed by RT-qPCR using primers and probes previously described (Corman et al., 2020; Wölfel et al., 2020): leader-specific primer sgLeadSARSCoV2-F CGATCTCTTGTAGATCTGTTCTC, E-Sarbeco-R primer ATATTGCAGCAGTACGCACACA and E-Sarbeco probe HEX-ACACTAGCCATCCTTACTGCGCTTCG-BHQ1. The protocol describing the procedure for the detection of SARS-CoV-2 is available on the WHO website (https://www.who.int/docs/default-source/coronaviruse/real-time-rt-pcr-assays-for-the-detection-of-sars-cov-2-institut-pasteur-paris.pdf?sfvrsn=3662fcb6_2).

#### Chest computed tomography and image analysis

Acquisition was done using a computed tomography (CT) system (Vereos-Ingenuity, Philips). Animals were anesthetized and put in a supine position. Body temperature, oxygen saturation and heart rate were monitored. The collimation of the CT detector was 64 × 0.6 mm, with a 120 kV tube voltage and around 120 mA intensity. The intensity was regulated by dose optimization tools (Dose Right, Z-DOM, 3D-DOM; Philips Healthcare). The reconstructed CT images had a slice thickness of 1.25 mm, with an interval of 0.25 mm. Lesions were defined, by internationally standard nomenclature, as ground glass opacity, crazy-paving pattern, consolidation or pleural thickening (Pan et al., 2020; Shi et al., 2020) (none, 0; GGO, 1; pleural thickening, 1; crazy-paving pattern, 3; consolidation, 3). Lesions and scoring were assessed in each lung lobe blindly and independently by two persons and final results were made by consensus. Overall CT scores include the lesion type, scored from 0 to 3 (None, 0; Slight, 1; Mild, 2; Severe, 3) and lesion volume, scored from 0 to 4 (None, 0; < 25% of the lobe, 1; 25 < x < 50% of the lobe, 2; 50 < x < 75% of the lobe, 3; 75 < x < 100% of the lobe; 4) and summed for each lobe.

#### ELISpot assays

IFNγ ELISpot assay of PBMCs was performed using the Monkey IFNγ ELISpot PRO kit (Mabtech Monkey IFNγ ELISPOT pro, #3421M-2APT) according to the manufacturer’s instructions. PBMCs were plated at a concentration of 200,000 cells per well and were stimulated with SARS-CoV-2 S protein at a final concentration of 5 μg/mL. Plates were incubated for 42 h at 37°C in an atmosphere containing 5% CO_2_, then washed 5 times with PBS and incubated for 2 h at 37°C with a biotinylated anti-IFNγ antibody. After 5 washes, spots were developed by adding 0.45 μm-filtered ready-to-use BCIP/NBT-plus substrate solution and counted with an automated ELISpot reader ELRIFL04 (Autoimmun Diagnostika GmbH, Strassberg, Germany). Spot forming units (SFU) per 1.0x10^6^ PBMCs are means of duplicates for each animal.

#### Viral sequencing

30 RNA samples from nasal swabs and bronchoalveolar lavage at day 3 and 5 were selected for sequencing. cDNA and multiplex PCR reactions were prepared following the ARTIC SARS-CoV-2 sequencing protocol v2 (Tyson et al., 2020). The inoculum was also sequenced. V3 primer scheme (https://github.com/artic-network/primer-schemes/tree/master/nCoV-2019/V3) was used to perform the multiplex PCR for SARS-CoV-2. All samples were run for 35 cycles in the two multiplex PCRs. Pooled and cleaned PCR reactions were quantified using QubitTM fluorometer (Invitrogen). The Ligation Sequencing kit (SQK-LSK109; Oxford Nanopore Technologies) was used to prepare the library following the manufacturer’s protocol (“PCR tiling of COVID-19 virus,” release F; Oxford Nanopore Technologies). 24 samples were multiplexed using Native Barcoding Expansion 1-12 and Native Barcoding Expansion 13-24 kits (EXP-NBD104 and EXP-NBD114; Oxford Nanopore Technologies). Two libraries of 24 samples were prepared independently and quantified by QubitTM fluorometer (Invitrogen). Quality control of two R9.4 flowcells (FLO-MIN106; Oxford Nanopore Technologies) was performed to assess pores viability. Next, 45 and 32 ng of library were respectively loaded as recommended by the manufacturer’s protocol. Sequencing was performed on a GridION (Oxford Nanopore Technologies) for 72 h with high-accuracy Guppy basecalling (v3.2.10). After sequencing, demultiplexing was performed using Guppy v4.0.14 with the option–require_barcodes_both_ends to ensure high quality demultiplexing. Reads were then filtered by Nanoplot v1.28.1 based on length and quality to select high quality reads. Then, reads were aligned on the SARS-CoV-2 reference genome NC_045512.2 using minimap2 v2.17. Primary alignments were filtered based on reads length alignment and reads identity. Finally, variant calling was performed with Longshot v0.4.1. Identified variants were then analyzed using in-house scripts. Variants absent from the inoculum but present in one or more samples were selected for further analysis.

### Quantification and statistical analysis

Details on the statistical analysis for the experiments can be found in the accompanying figure legends. Statistical significance between groups is indicated in the figures by stars: ^∗^, p < 0.05; ^∗∗^, p < 0.01; ^∗∗∗^, p < 0.001; ^∗∗∗∗^, p < 0.0001. In addition, statistical significance between viral loads in the control and vaccinated groups are also indicated in the Results section by p values. Medians are plotted for all experiments and comparisons between two groups were made using a Mann-Whitney U test (Excel 2016, GraphPad Prism 8.0/7.0). For mouse experiments 8 animals were used up to week 6, when 2 animals were euthanized for preliminary assessment of (N)Ab titers. Due to low sample volumes the eight mouse samples at week −1 were randomly paired and pooled into 4 samples. The rabbit experiments were performed using 5 animals. In the macaque experiments, 6 animals were used in the vaccinated group and 4 animals were used in the control group.

## Supplemental information

Document S1. Tables S1 and S2

Table S3. Viral variants identified by viral sequencing in control and vaccinated cynomolgus macaques after challenge, related to Figure 6
