# Supplementary material for: Two-component spike nanoparticle vaccine protects macaques from SARS-CoV-2 infection
Source: Cell. 2021 Mar 4;184(5):1188–1200.e19. doi: 10.1016/j.cell.2021.01.035 (PMC7834972; doi:10.1016/j.cell.2021.01.035)
Supplement: Document S1. Tables S1 and S2 [file mmc1.pdf]

Cell, Volume *184*

Supplemental information

Two-component spike nanoparticle vaccine protects macaques from SARS-CoV-2 infection

Philip J.M. Brouwer, Mitch Brinkkemper, Pauline Maisonnasse, Nathalie Dereuddre-Bosquet, Marloes Grobben, Mathieu Claireaux, Marlon de Gast, Romain Marlin, Virginie Chesnais, Ségolène Diry, Joel D. Allen, Yasunori Watanabe, Julia M. Giezen, Gius Kerster, Hannah L. Turner, Karlijn van der Straten, Cynthia A. van der Linden, Yoann Aldon, Thibaut Naninck, Ilja Bontjer, Judith A. Burger, Meliawati Poniman, Anna Z. Mykytyn, Nisreen M.A. Okba, Edith E. Schermer, Marielle J. van Breemen, Rashmi Ravichandran, Tom G. Caniels, Jelle van Schooten, Nidhal Kahlaoui, Vanessa Contreras, Julien Lemaître, Catherine Chapon, Raphaël Ho Tsong Fang, Julien Villaudy, Kwinten Sliepen, Yme U. van der Velden, Bart L. Haagmans, Godelieve J. de Bree, Eric Ginoux, Andrew B. Ward, Max Crispin, Neil P. King, Sylvie van der Werf, Marit J. van Gils, Roger Le Grand, and Rogier W. Sanders

Table S1 SARS-CoV-2 pseudovirus and authentic virus serum neutralization (ID50s) in BLAB/c mice and rabbits, related to figure 3.

Pseudovirus neutralization

	Animal ID	Week -1/0	Week 6	Week 14
Mice	1	<100*	75063	n.d.
	2		25593	63015
	3	<100*	2588	231100
	4		23399	63418
	5	<100*	2992	n.d.
	6		8179	17235
	7	<100*	14035	35046
	8		19548	18749
Rabbits	1	<100	68298	34813
	2	<100	41968	76139
	3	<100	41162	135128
	4	<100	77885	152796
	5	<100	90884	186147

Authentic virus neutralization

	Animal ID	Week 14
Mice	1	n.d.
	2	6700
	3	9716
	4	4651
	5	n.d.
	6	1649
	7	3478
	8	3068
Rabbits	1	4514
	2	28180
	3	15110
	4	10138
	5	30851

n.d.	Not determined
	<100
	101 - 1,000 ID50
	1,001 - 10,000 ID50
	10,001 - 100,000 ID50
	>100,001 ID50

*Samples were pooled

Table S2. SARS-CoV-2 pseudovirus and authentic virus neutralization titers (ID50s) in cynomolgus macaques, related to figure 5.

Pseudovirus neutralization

	Animal ID	Week 0	Week 2	Week 4	Week 6	Week 8	Week 10	Week 12	Week 14	Week 15	Week 18
Control macaques	MF1	n.d.	n.d.	n.d.	n.d.	n.d.	n.d.	<100	112	452	299
	MF2	n.d.	n.d.	n.d.	n.d.	n.d.	n.d.	<100	6583	1553	311
	MF3	n.d.	n.d.	n.d.	n.d.	n.d.	n.d.	<100	1818	1442	571
	MF4	n.d.	n.d.	n.d.	n.d.	n.d.	n.d.	<100	381	344	<100
Vaccinated macaques	MF5	<100	162	251	11340	9179	3043	30766	8212	6615	3782
	MF6	<100	912	<100	2981	1324	1469	10720	10384	4415	2690
	MF7	<100	584	<100	10171	6646	2780	29198	12679	10476	5322
	MF8	<100	599	<100	5087	3894	3150	30080	20526	4507	2762
	MF9	<100	183	<100	22450	30300	22328	23523	32909	11352	5680
	MF10	<100	<100	2515	7558	2991	1101	17128	19730	4376	8544

Authentic virus neutralization

	Animal ID	Week 0	Week 6	Week 12
Control macaques	MF1	n.d.	n.d.	n.d.
	MF2	n.d.	n.d.	n.d.
	MF3	n.d.	n.d.	n.d.
	MF4	n.d.	n.d.	n.d.
Vaccinated macaques	MF5	<20	792	4190
	MF6	<20	166	1239
	MF7	<20	2155	5095
	MF8	<20	1629	1913
	MF9	<20	3040	3694
	MF10	<20	1372	4851

Legend (cell colour):
- <100
- 101 - 1,000 ID50
- 1,001 - 10,000 ID50
- 10,001 - 100,000 ID50
- >100,001 ID50

Authentic virus neutralization post challenge

	Animal ID	Week 12	Week 15	Week 18
Control macaques	MF1	<20	813	94
	MF2	<20	1110	32
	MF3	<20	444	137
	MF4	<20	<20	<20
Vaccinated macaques	MF5	2519	2528	2469
	MF6	2456	3963	2840
	MF7	11069	8586	5102
	MF8	8782	3020	665
	MF9	8606	11036	2842
	MF10	6345	3321	1190
